# Split-Ring Structured All-Inorganic Perovskite Photodetector Arrays for Masterly Internet of Things

**DOI:** 10.1007/s40820-022-00961-y

**Published:** 2022-11-29

**Authors:** Bori Shi, Pingyang Wang, Jingyun Feng, Chang Xue, Gaojie Yang, Qingwei Liao, Mengying Zhang, Xingcai Zhang, Weijia Wen, Jinbo Wu

**Affiliations:** 1 Materials Genome Institute, Shanghai University, Shanghai, 200444 People’s Republic of China; 2 Zhejiang Laboratory, Hangzhou, 311100 People’s Republic of China; 3 HKUST Shenzhen-Hong Kong Collaborative Innovation Research Institute, Futian, Shenzhen, People’s Republic of China; 4 School of Engineering, Massachusetts Institute of Technology, Cambridge, MA 02139 USA; 5 School of Engineering and Applied Sciences, Harvard University, Cambridge, MA 02138 USA; 6 Department of Physics, Shanghai University, Shanghai, 200444 People’s Republic of China; 7 The Advanced Material Thrust, The Hong Kong University of Science and Technology (Guangzhou), Guangzhou, People’s Republic of China

**Keywords:** Split-ring, Dewetting, Perovskite photodetector array, Human–machine interface, Gesture recognition

## Abstract

Photodetectors with long detection distances and fast response are important media in constructing a non-contact human–machine interface for the Masterly Internet of Things (MIT). All-inorganic perovskites have excellent optoelectronic performance with high moisture and oxygen resistance, making them one of the promising candidates for high-performance photodetectors, but a simple, low-cost and reliable fabrication technology is urgently needed. Here, a dual-function laser etching method is developed to complete both the lyophilic split-ring structure and electrode patterning. This novel split-ring structure can capture the perovskite precursor droplet efficiently and achieve the uniform and compact deposition of CsPbBr_3_ films. Furthermore, our devices based on laterally conducting split-ring structured photodetectors possess outstanding performance, including the maximum responsivity of 1.44 × 10^5^ mA W^−1^, a response time of 150 μs in 1.5 kHz and one-unit area < 4 × 10^–2^ mm^2^. Based on these split-ring photodetector arrays, we realized three-dimensional gesture detection with up to 100 mm distance detection and up to 600 mm s^−1^ speed detection, for low-cost, integrative, and non-contact human–machine interfaces. Finally, we applied this MIT to wearable and flexible digital gesture recognition watch panel, safe and comfortable central controller integrated on the car screen, and remote control of the robot, demonstrating the broad potential applications.

**Supplementary Information:**

The online version contains supplementary material available at 10.1007/s40820-022-00961-y.

## Highlights

- The split-ring topography is studied systematically from wettability, evaporation assembly to optoelectronic devices.
- An efficient dual-function laser etching scheme has been developed to fabricate the split-ring lyophilic pattern and the lateral electrode array simultaneously.
- A non-contact human-machine interface based on CsPbBr_3_ perovskite photodetector arrays has been successfully applied to wearable devices, automobile displays, robot remote control.

**Supplementary Information:**

The online version contains supplementary material available at 10.1007/s40820-022-00961-y.

## Graphical abstract

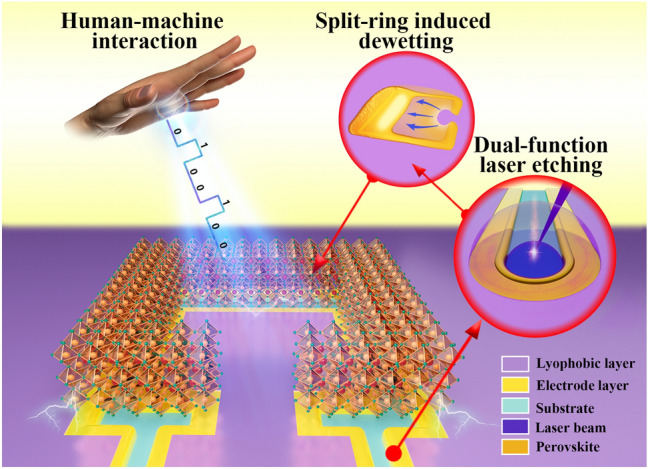

## Introduction

Intelligent perception and control through human–machine interaction often rely on the acquisition and processing of signals from acoustic, optical, electrical, and mechanical sensors [1–6]. Therefore, an intelligent, fast, and friendly interface is essential for information transmission and action execution between humans and machines, while humans often input signals into machines by pressing mechanical buttons or touching electronic screens, which makes mechanical wear and fatigue inevitable for interactive interfaces. Moreover, these physical contacts are very likely to become a medium for the transmission of bacteria, viruses, and harmful substances between different populations in special environments such as hospitals and laboratories. Consequently, non-contact sensors (such as photodetectors, humidity sensors, and electrostatic sensors) are becoming the development trend for human–machine interfaces [7–11]. Among them, photodetectors, which can convert optical signals into electrical signals, have been widely used in image sensing, optical communication, and other applications [12–16]. Non-contact optoelectronic interfaces, which are composed of photodetector arrays, perceive the motion of a distant object through shadow detection. Such devices have gradually become innovative human–machine interactions due to their advantages of long detection distances and fast response time.

Various types of optoelectronic materials have been reported to fabricate photodetectors in the past, such as silicon semiconductors, and organic polymer. But they usually have complicated structures and require high-temperature or complex fabrication processes, leading to high cost [17, 18]. Under the trend of miniaturization, arraying and flexibility of devices, simple and efficient fabrication processes can meet greater commercial needs. Metal halide perovskite materials, which are considered as suitable candidate for optoelectronic devices [19], due to their excellent optoelectronic properties, such as high carrier mobility and high absorption coefficients [20, 21]. Furthermore, compared with organic–inorganic hybrid perovskites, all-inorganic perovskites exhibit better long-term stability in the environment due to higher moisture and oxygen resistance [22–26]. However, the devices made of all-inorganic perovskite films perform inconsistently due to the difficulty associated with controlled nucleation and crystallization process [25, 27]. On the other hand, although many researchers have applied perovskite materials to miniaturized, arrayed and integrated photodetectors, including photoconductors [28–32], photodiodes [33–36], and phototransistors [37, 38], the geometric constraints of these optoelectronic material often require photolithography and mask thermal evaporation to complete patterning and arraying [39]. Therefore, the high cost and complex fabrication process have limited the wider commercial application of optoelectronic devices based on perovskite materials [40, 41].

**Fig. 1:**
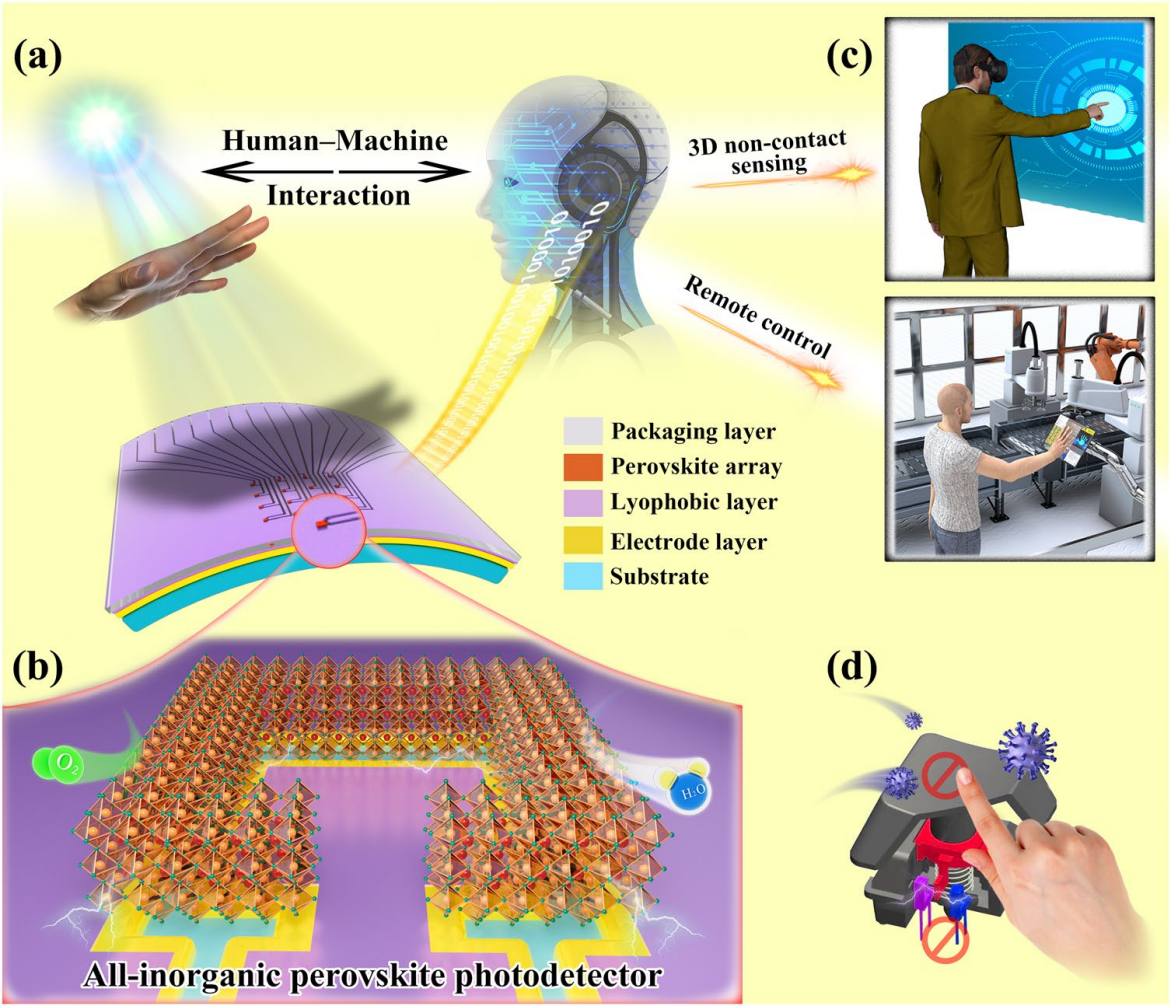
Schematic of CsPbBr_3_ photodetector array-based human–machine interaction process for MIT. **a** A schematic diagram of the structure of the human–machine interface. The device is composed of a packaging layer, a lyophilic layer, a perovskite film array, an electrode layer, and a substrate. **b** Schematic diagram of the split-ring structured perovskite photodetector. **c** Applications of device in human–machine interaction. **d** This durable and sterile interface versus the mechanical button with virus

In this study, we reported an all-inorganic CsPbBr_3_ split-ring structured photodetector (SRP) array for non-contact human–machine interaction (Fig. 1). We developed a dual-function laser etching scheme that can simultaneously complete the patterning of lyophilic layer and electrodes, which greatly reduced the cost and processing steps, and provided new insights into the preparation of laterally structured devices. In addition, we achieved the efficient directional transport of the perovskite precursor solution to the electrodes through a novel split-ring lyophilic pattern and realized the high crystallinity of CsPbBr_3_ perovskite films by reducing the deposition area by 62%. Finally, we applied a high-performance split-ring structured photodetector array to a non-contact human–machine interface for Masterly Internet of Things**,** achieving digital gesture recognition in a flexible wearable device and innovatively developing a three-dimensional (3D) gesture motion sensing scheme that is integrated into the display. This scheme allows humans to interact more flexibly with the intelligent system by using 3D gestures. We also successfully controlled a robot remotely through this interface, which verifies the multifunctional development potential of the device.

## Experimental Section

### Materials

All chemical reagents were used as received and without further purification, including cesium bromide (CsBr, 99.9%, Aladdin Reagent, Shanghai, China), lead bromide (PbBr_2_, 99.9%, Aladdin Reagent, Shanghai, China), dimethyl sulfoxide (DMSO, 99.9%, Aladdin Reagent, Shanghai, China), and 1H,1H,2H,2H-Perfluorooctyltriethoxysilane (POTS, 98%) were purchased from Sigma-Aldrich, Co. Indium tin oxide-glass (ITO-glass, type: YK-ITO-Y11-10, ITO thickness: 180 nm, sheet resistivity: 10 Ω) was purchased from Suzhou ShangYang Solar Technology Co., Ltd.

### Device Fabrication

#### Preparation Process of Lyophilic–lyophobic Pattern on the Substrate

ITO-glass was, respectively, ultrasonically cleaned in ethanol and deionized water for 5 min, and the surface of ITO-glass was further cleaned for 2 min with Plasma Cleaner (PDC-002-HP Plasma Cleaner) after it was fully dried. Lyophilic groups were generated on the treated substrate, which can increase the wettability of the substrate. The substrate and an open container containing 2 μL PTOS were placed in an airtight container with the oven (BINDER GmbH FD56) at 120 °C for 2.5 h. The surface of the substrate was covered with POTS molecular layer and had good lyophobic properties. Finally, the substrate was etched by nanosecond pulse laser (Bellin-SP350-10, repetition frequency 50 kHz, average power 1.045 W, and wavelength 355 nm) to form a lyophilic–lyophobic pattern array.

#### Fabrication of CsPbBr_3_ Array

PbBr_2_ and CsBr were dissolved in polar solvent DMSO at a molar ratio of 1:1, heated and dispersed ultrasonically by an ultrasonic cleaning machine (Elmasonic Easy 60 H) for 4 h to synthesize 0.4 mol L^−1^ CsPbBr_3_ precursor solution. The coating process used our independently developed device: the glass brush was placed vertically above the base at a height of 10 μm, the gap between the brush and the substrate was filled with 10 μL of precursor solution, and the coating was carried out at a uniform speed of 150 mm min^−1^ under the drive of the stepper motor. At this time, a high-throughput precursor array will be formed at the lyophilic pattern on the substrate. Subsequently, the substrate was quickly transferred to a heating plate (RCT Basic S025) at 60 °C, and the high crystallinity perovskite film array was formed by heating assisted evaporation of precursor droplets. The above process was completed in a glove box with an ambient temperature of 25 °C and a relative humidity of 30%. Finally, the samples were placed in a vacuum oven (BluePard DZF-6050) at 80 °C for 8 h to improve the crystal quality of perovskite films.

#### Fabrication of Photodetector Array Device

Several ITO conductive channels were formed on the surface of the substrate by laser etching. One end of the channel extended to the edge of the substrate to facilitate the connection of external circuits. The other end of the channel was connected to the gap in the split ring pattern, isolating the inside and outside of the pattern from each other, thus forming a lateral structure of the dual-electrode device.

### Characterizations

#### Morphology and Structure Characterization

The intrinsic contact angles and surface adhesion energy of substrate with different surface properties were measured by contact angle analyzer (Kruss-DSA25). The dewetting and crystallization processes of microdroplet at high time resolution were captured by microscope (Olympus IX73) and high-speed camera (PHANTOM MIRO R311). The optical microscopic image of the CsPbBr_3_ film units and arrays were obtained by a microscope (LEICA DFC7000T). The confocal micrographs of fluorescein sodium salt/glycerol taken by laser scanning confocal microscope (Olympus FV3000). The morphology, element distribution and cross section profile of the etch lines on the substrate surface and CsPbBr_3_ films were, respectively, characterized by field emission scanning electron microscopy (Hitachi, SU-8230), energy dispersion spectrometer (OXFORD Ultim Extreme) and surface profilometer (Alpha-Step D-300), respectively. X-ray diffraction spectrum of CsPbBr_3_ were obtained using X-ray diffractometer (PANalytical Empyrean Alpha 1).

#### Optical and Optoelectronic Characterization

The absorption and photoluminescence spectrum of CsPbBr_3_ films were, respectively, measured by UV–Visible absorption spectrometer (PERKIN-ELMER Lambda750) and fluorescence spectrometer (Edinburgh FS-5 UK); the fluorescence lifetime of CsPbBr_3_ films at excitation wavelength of 365 nm was measured by time-resolved fluorescence spectrophotometer (Edinburgh Photonics Mini-Tau). The performance of the photodetectors was characterized by a customized testing platform and all the tests were carried out in ambient condition. We use an arbitrary waveform generator (TeKtronix AFG3251C) as a voltage source to control the LED lamp (CREE XPE2) and laser lamp (Thorlabs CPS532). The light power density is calibrated by power meter (Newport 843-R). The voltage ampere characteristic curve of the photodetectors was tested with semiconductor parameter instrument (Keithley 4200). The response time and stability of the photodetector were tested with oscilloscope (KEYSIGHT DSOX1202A). A data acquisition system (multifunctional I/O device NI PCI-6259 and junction box NI SCB-68A) was used to measure the photocurrent value of the photodetector array in multi-channel real-time.

## Results and Discussion

### Mechanism of Split-Ring Topography-Assisted Dewetting

**Fig. 2:**
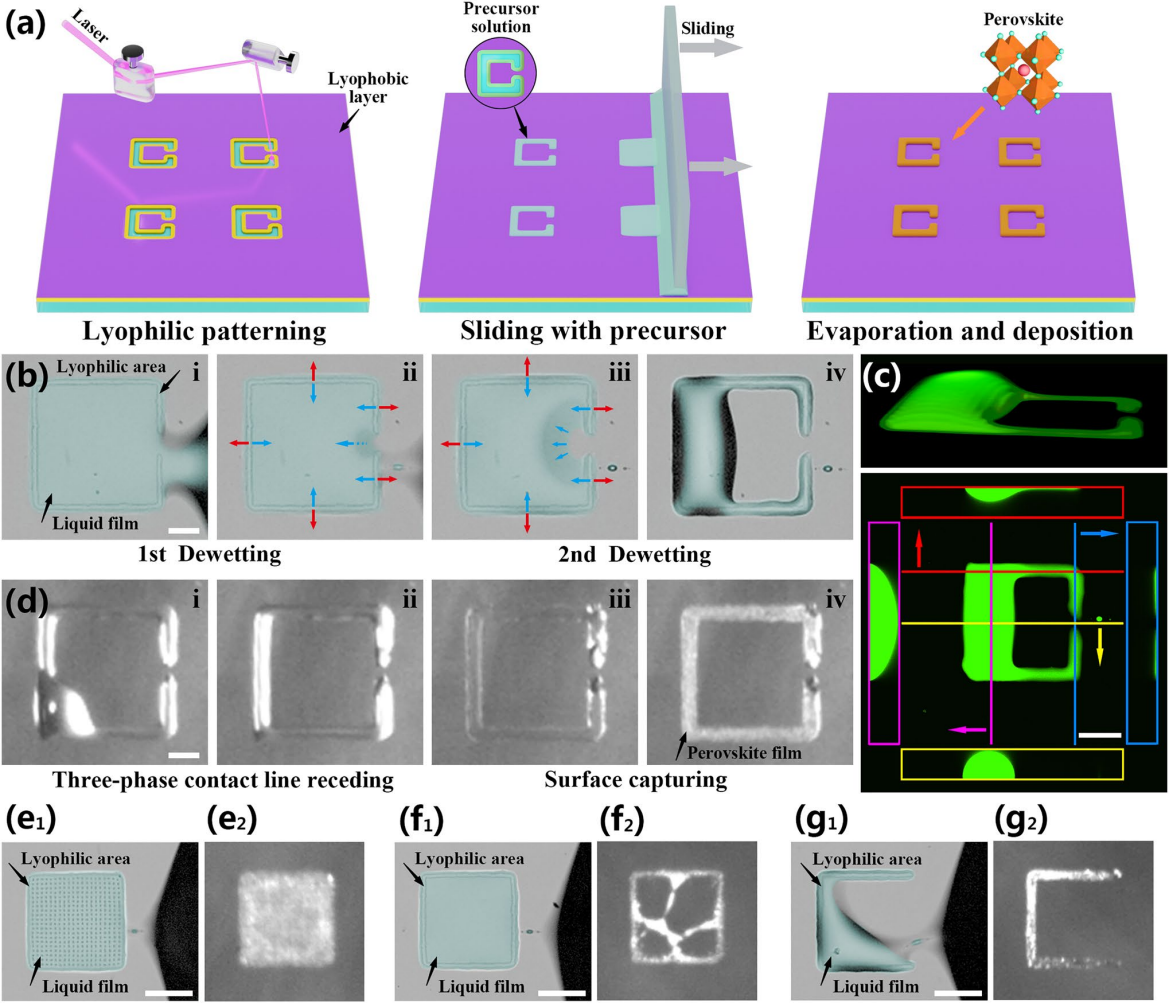
**a** Schematic diagram of the preparation process of the photodetector array based on the split-ring structured perovskite film. **b** The perovskite precursor solution dewetting processes captured by a high-speed camera (blue and red arrows indicate surface tension and pinning force, respectively, scale bar: 50 μm). **c** Themorphology of a glycerol split-ring droplet taken by Confocal microscope after dewetting process (scale bar: 80 μm). **d** The evaporation process of precursor solution captured by a high-speed camera on the hot plate (scale bar: 50 μm). **e–g** The dewetting and deposition results of a fully lyophilic square pattern, a square ring pattern without opening, the split-ring pattern, and a trilateral pattern (scale bar: 100 μm)

A schematic diagram of the perovskite array preparation process is shown in Fig. 2a, lyophilic patterning by laser etching on a lyophobic substrate modified by 1H,1H,2H,2H-perfluorooctyltriethoxysilane (POTS), sliding the perovskite precursor solution, and evaporation self-assembly of the crystalline film. Among these steps, the lyophilic–lyophobic patterning of the substrate is a key step for the preparation of perovskite film arrays. This pattern can assist the generation of the droplet array and the efficient directional transport of precursor solution by dewetting, ensuring the dense and uniform deposition of particles in the perovskite film.

Discontinuous dewetting refers to the process in which the solid–liquid interface is discontinuously replaced by the solid–gas interface, which is often employed during the liquid coating on the lyophilic–lyophobic patterned substrate to generate droplet arrays in a high-throughput manner [42, 43]. In this dewetting process, a split-ring-structure lyophilic pattern assisted the droplet unit to dewet again inside the pattern, to achieve the directional transport process of the precursor solution. The outer boundary and the gap of the pattern determined the first and second dewetting processes. We recorded these two rapid dewetting processes using CsPbBr_3_ precursor solution captured by a high-speed camera (Fig. 2b and Video S1). Under the external force, the advancing mother-solution continuously wetted and dewetted on the substrate until the retreated three-phase contact line (TCL) met the boundary of the pattern, and solution was pinned by the lyophilic surface to form a liquid bridge. The liquid bridge gradually became thinner and narrower under the continuous pulling until it broke up in an instant to leave a son-droplet in the lyophilic pattern (Fig. 2b_iv_).

The time intervals of Fig. 2b_i_ to 2b_ii_ and Fig. 2b_ii_ to 2b_iii_ are both 0.5 ms. It is worth noting that a small satellite droplet first appeared in the liquid bridge in Fig. 2b_ii_, which indicated that the liquid bridge was breaking up at this moment. When the liquid bridge broke up, the TCL shrank along the outer boundary of the lyophilic pattern and contacted the lyophobic gap. This split-ring structured liquid film was in a metastable stage due to the imbalance between the pinning force and surface tension (Fig. 2b_iii_). Therefore, the TCL of the liquid film retracted continuously to the lyophobic interior of the split-ring pattern and became a droplet, which manifested as the second dewetting process (Fig. 2b_iii_ to 2b_iv_, 2 ms). The static contact angle between the CsPbBr_3_ precursor solution and the POTS-modified substrate was approximately 84° ± 3°, and the laser-etched area was 26° ± 4°, indicating that the wettability of the substrate surface differed significantly before and after laser etching (Fig. S1).

To prove that the second dewetting process was spontaneous, we calculated the adhesion energy between the solvent dimethyl sulfoxide (DMSO) and the regions with different wettability on the substrate. In our experiment, the adhesion energy *I*_*SL*_ was calculated with the Fox equation:

1 $${I}_{SL}=2[({\sigma }_{L}^{D}{)}^{1/2}({\sigma }_{S}^{D}{)}^{1/2}+({\sigma }_{L}^{P}{)}^{1/2}({\sigma }_{S}^{P}{)}^{1/2}]$$

wherein ${I}_{SL}$ is the adhesion energy between a liquid and a solid surface per unit area, ${\sigma }_{L}^{D}$ is the dispersive component of the surface tension of the wetting liquid, ${\sigma }_{L}^{P}$ is the polar component of the surface tension of the wetting liquid, ${\sigma }_{S}^{D}$ is the dispersive component of the surface energy of the solid, and ${\sigma }_{S}^{P}$ is the polar component of the surface energy of the solid. This equation uses a dispersion component and a polar component to describe the surface energy of a solid. The adhesion energy in the lyophobic region was calculated to be 51.8 mN m^−1^. The lyophilic region was the trench region of ITO etched by laser, with an adhesion energy of 95.9 mN m^−1^. Therefore, the adhesion force of the lyophilic zone to the DMSO was greater than the lyophobic one, which supported the claimed ability of the second dewetting process to occur inside the split-ring pattern. A more detailed formula derivation process and relevant data are shown in Table S1.

To characterize the 3D structure of the split-ring droplet, the 3D images of the droplet after dewetting were taken by laser confocal scanning microscopy (LCSM). We chose 40 wt% glycerol instead of DMSO as the solvent to avoid quick evaporation during optical sectioning. Detailed properties of glycerol and DMSO can be found in Table S2. The 3D projection, vertical and cross-sectional views of glycerol droplet dyed with fluorescein sodium salt are shown in Fig. 2c. This split-ring structured droplet can be viewed as a connected liquid puddle of a large droplet, crossing the lyophilic and lyophobic regions and forming a high local contact angle, and two extended liquid films on the lyophilic region forming a small local contact angle.

The deposition process of our split-ring patterned liquid could be affected by multiple mechanisms. Figure 2d and Video S2 showed the evaporation and self-assembly crystallization process of the precursor droplet array at 60 °C and 30% humidity under high-speed imaging. The first stage of this process occurred during the initial stage of heating, when the substrate temperature gradually increased, the TCL of the large droplet steadily retracted to the lyophilic region. In the second stage, the precursor droplets reached the critical concentration for nucleation, and then gradually precipitated perovskite crystal nuclei and nanoparticles. Due to the pinning of the lyophilic pattern on the droplet, the TCL of the droplet could not contract further at this time. With the continuous evaporation of the solvent by heating, the shrinkage rate of the gas–liquid interface of the solution film exceeded the average diffusion rate of the perovskite nanoparticles, and the precipitated nanoparticles were captured by the rapidly dropping gas–liquid interface, forming a semisolid layer with a uniform grain distribution. After the solvent had completely evaporated, a dense deposition of particles in the perovskite film was achieved. Figure S2 further compared and discussed the evaporation mechanism and the crystal deposition morphology of the split-ring pattern under room temperature and heating conditions.

To further study the dewetting dynamics, we compared the dewetting and deposition results of four lyophilic patterns with similar outer contours including a fully lyophilic square pattern, a square ring pattern without opening, the split-ring pattern, and a trilateral pattern in Fig. 2e–g. Meanwhile, their dewetting processes, confocal micrographs and evaporation processes are shown in Figs. S3-S5. The confocal micrographs reflect the three-dimensional topography of those four patterns. The liquid volume of those four patterns are calculated by stacking the sectional images. The droplet volumes of the fully lyophilic square, the square ring, the split-ring and the trilateral pattern are 3.4, 3.4, 2.8, and 0.9 nL, respectively. Compared to the fully lyophilic and square ring patterns, the split-ring pattern can maintain 82% amount of liquid. The crystallized film from the fully lyophilic square pattern with a large deposition area shows poor compactness (Fig. S6). Due to the interior of the square ring pattern is lyophobic, liquid film rupture or dewetting may occur during evaporation, resulting in a random deposition. With the lyophilic topography structure changing from ring to split-ring, the deposition area is reduced greatly to 38% of the whole pattern (that is lyophilic area) while droplet volume maintained almost the same. To explore the influence of split-ring structure, the deposition results of square and circular rings with different gap widths and diameters or side lengths are shown in Fig. S7. Through the optimization and comparison, we conclud that the split-ring structure with a length and gap of approximately 200 and 30 μm can achieve the best compactness of the perovskite film by surface capturing of the droplet without any passivation agent or spatial template, which provides a prerequisite for the excellent optoelectronic performance of the device.

### High-Throughput Fabrication of Split-ring-structure Perovskite Arrays

**Fig. 3:**
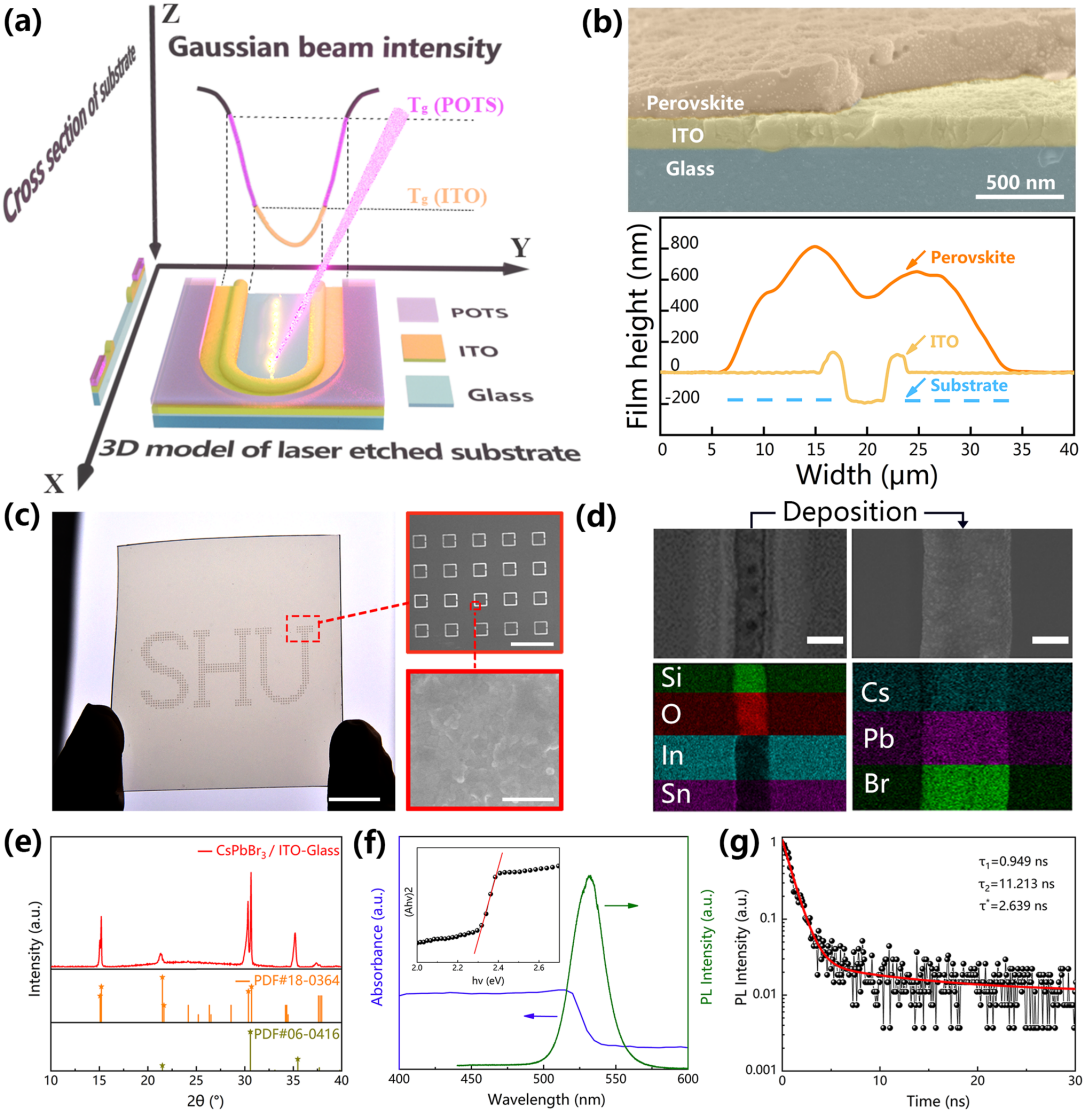
**a** Schematic diagram of the mechanism of laser etching. The XY and XZ planes showed the 3D and cross-sectional views of the etching effect, respectively. The YZ plane showed the Gaussian distribution of the laser beam intensity. **b** The cross section SEM image of the perovskite-deposited ITO-glass. The surface profile of the ITO-glass after laser etching and the perovskite-deposited ITO-glass shown in the bottom graph. **c** An optical image of the perovskite film array patterned as “SHU” on the glass substrate (scale bar: 1 cm). The inset showed the SEM image of part of the perovskite array (scale bar: 600 μm) and enlarged view of film (scale bar: 1 μm). **d** The top and bottom pictures severally were SEM and EDS mapping images of the substrate before (left) and after (right) perovskite deposition. The scale bar is 8 μm. **e** XRD spectra of the perovskite array. **f** The PL and absorption spectra of CsPbBr_3_. The inset showed the spectrum for calculating the optical band gap. **g** A time-resolved PL spectroscopy curve of the CsPbBr_3_ arrays

Here, a dual-function laser etching method is proposed for the one-step fabrication of the patterned lyophilic–lyophobic surface and electrode array on the substrate, then the split-ring pattern is used to deposit the perovskite film on the ITO dual-electrodes. A schematic diagram of the mechanism of laser etching is shown in Fig. 3a. The YZ plane shows the typical Gaussian distribution of the intensity of the laser beam (note: the energy increases down the Z-axis), and the XY and XZ planes show the 3D and cross section of the laser-etched substrate severally. When a Gaussian laser beam acts on the surface of the substrate, the energy at the center of the laser spot is sufficiently intense to vaporize the ITO electrode layer; at the same time, the edge of the laser spot reduces the lyophobic effect of the adjacent POTS layer through a photothermal effect, forming a heat-affected zone. Interestingly, due to the width of the heat-affected zone is always significantly larger than that of the etched zone, the area that can be wetted by the precursor solution can completely cover the ITO trench. The perovskite film with densely packed particles can cross the two sides of the trench from ITO etching laterally to form a dual-electrode device after evaporation and crystallization (Fig. S8b and the top of Fig. 3b). The depth of the ITO trench by laser etching is 180 nm, which is consistent with the thickness of the ITO film. The schematic diagram and result of the surface profile test are shown in Fig. S8a and the bottom of Fig. 3b, respectively. In addition, the surface height after perovskite deposition is approximately 600 nm, and the perovskite film completely cover both sides of the trench from ITO etching. Figure S9 depicts the effect of laser power density on the etching width of the ITO and POTS. As the laser power density increases, the thermal effect at the laser spot also enhences. The use of excessive laser power density will cause damage to the glass substrate and produce a melting zone of ITO, resulting in a rough surface. In contrast, the use of insufficient laser power density cannot fully evaporate the ITO conductive layer, resulting in the failure of the ITO dual-electrodes. Therefore, we found a appropriate laser not only to effectively etch the ITO conductive layer but also to thermally vaporize the relatively wide POTS lyophobic layer, which is conducive to the effective deposition of perovskite films.

The letters “SHU” are composed of an array of split-ring structured perovskite films, and the insets show locally zoomed-in SEM images of the array (Fig. 3c). The area of perovskite unit less than 200 μm^2^, at this array density, a 1 cm^2^ substrate can accommodate 900 units. The average transmittance of the sample in the visible light range exceeds 83% (Fig. S10). The excellent optical transparency of the perovskite array allows it to be easily integrated on the surface of glass or screen and to act as a display without blocking the background image, which further expands the application of transparent sensors. In Fig. 3d, the major elemental distribution on the etched line are Si and O, while outside the etched line are In and Sn, indicating that the etched area is the exposed glass substrate due to the complete vaporization of the ITO layer in this area. Cs, Pb, and Br are uniformly distributed in the deposited crystalline film, and the perovskite film completely cover both sides of the trench from ITO etching. This result is in perfect agreement with the surface profile data in Fig. 3b. The CsPbBr_3_ film after annealed at 80 °C exhibit evident double diffraction peaks at 15° and 30° in X-ray diffractometer (XRD) spectrum (Fig. 3e). In addition, the characteristic peak of In_2_O_3_ in the cubic crystal orientation can be clearly observed at 35.5°, indicating that no foreign impurities are introduced during the entire preparation process. The absorption, photoluminescence (PL) and calculated optical band gap spectrum of the CsPbBr_3_ perovskite film in Fig. 3f show an absorption cutoff at ≈ 540 nm and the bandgap is calculated about 2.3 eV. The perovskite exhibits a photoluminescence (PL) emission peak at ≈ 530 nm with a full width at half maximum of ≈ 25.8 nm suggests a high color purity. The carrier lifetime can be calculated by measuring the fluorescence kinetic parameters of the CsPbBr_3_ film. After double exponential fitting of the fluorescence decay curve, the short lifetime *τ*_1_  is  0.949 ns and the long lifetime *τ*_2_  is  11.213 ns of the CsPbBr_3_ film, which correspond to the surface recombination and bulk recombination of electrons and holes after excitation (Fig. 3g).

### Photoelectric Properties of CsPbBr_3_ Split-ring-structure Photodetector

**Fig. 4:**
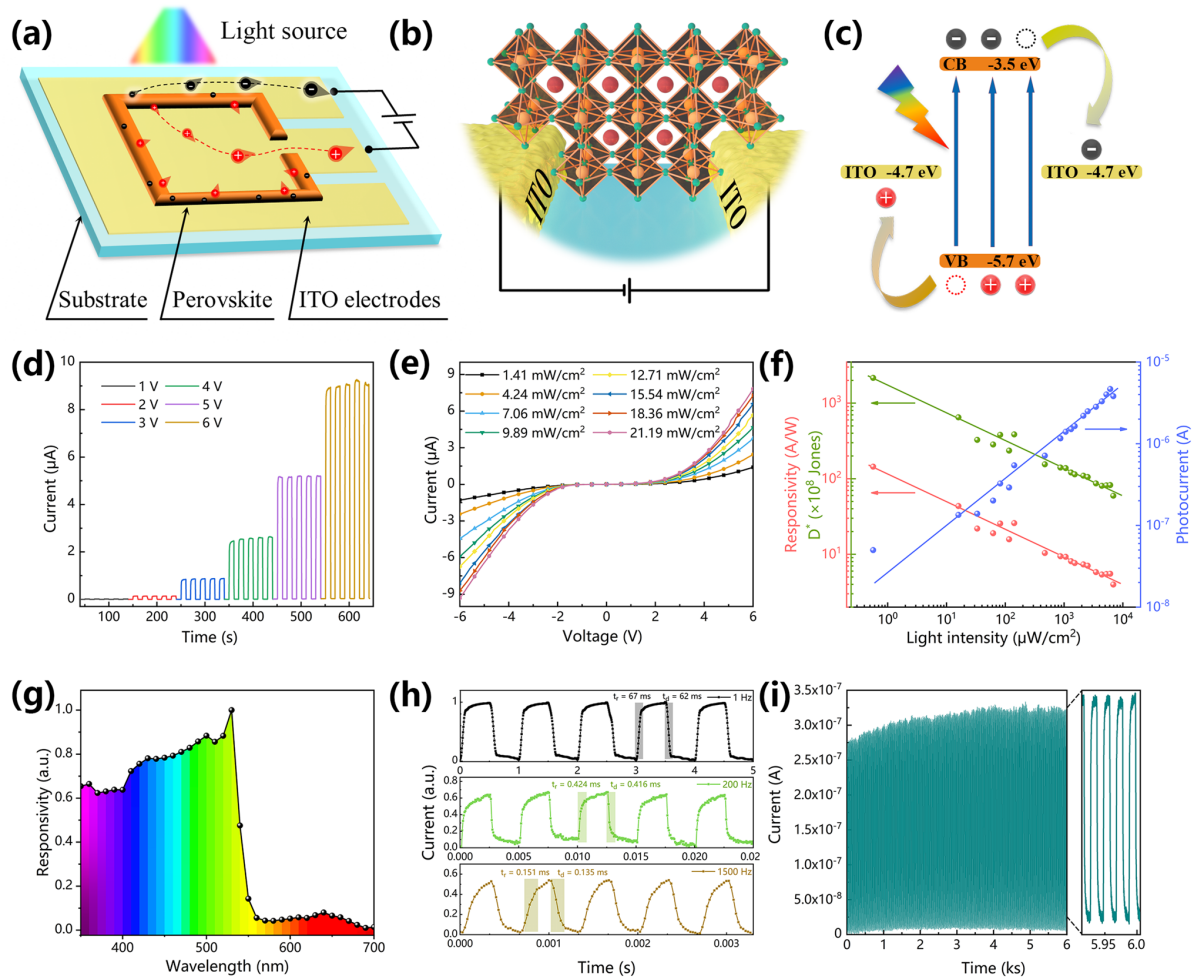
Photodetection performance of individual perovskite photodetector on a glass substrate. **a** Schematic diagram of the lateral structure and working mechanism of an SRP. **b** Schematic diagram of partial enlargement of SRP. **c** The energy-band alignment of SPR. **d** Current–time (I–t) curves of the perovskite photodetector under different bias voltages when irradiated with a 450 nm pulsed light source. **e** I-V curves of the photodetector under the irradiation of a 450 nm light source at different light powers.** f** Dependence of the responsivity, the D* and the photocurrent on the light intensity at the 520 nm. **g** A normalized responsivity curve of the photodetector under different illumination wavelengths (350 – 700 nm). **h** I-t curves at a 6 V bias and different on/off frequencies of 520 nm light sources. **i** On/off switching stability test of the device at a 3 V bias and 520 nm pulsed light source at a frequency of 0.05 Hz

Compared with the vertical structure, the lateral structure has advantages of easy preparation and integration in dual-electrode devices. Laser etching is used to close and appropriately extend the split-ring area where ITO is etched under the perovskite film to form two independent ITO electrodes inside and outside the pattern. The working mechanism diagram of the SPR and partial enlargement is shown in Fig. 4a–b. In contrast to the traditional symmetric electrode structure, the photogenerated current of this photodetector flows laterally inside and outside the pattern, and the conduction distance is only the width of the laser-etched area. This conduction mode greatly narrows the current transmission distance and fully utilizes the perovskite photoactive layer. Figure 4c shows the energy-band alignment of the photodetector. Under irradiation, the CsPbBr_3_ crystal generates electron–hole pairs, which are collected by the ITO electrodes at both ends under the driving force of an external bias voltage to form a photogenerated current. Since the photogenerated carriers will drift under a bias voltage and the drift velocity of the carriers is proportional to the magnitude of the photocurrent, the photocurrent of the device increases as the applied bias voltage increases (Fig. 4d). Figure S11 shows the current–voltage (I–V) curves of the photodetector measured under dark and a light intensity of 10 mW at 520 nm. The maximum on/off ratio and the dark current of this device measured at 2.3 V are 8.2 × 10^3^ and 2.03 × 10^–11^ A, respectively. Figure 4e presents the I–V curves of the photodetector under 450 nm irradiation with different light intensities. Due to the Schottky barrier at the contact interface between the CsPbBr_3_ perovskite film and the ITO electrode, all the curves exhibite asymmetric rectification. Under the same bias voltage, the number of photogenerated carriers is proportional to the absorbed photon flux. Therefore, increasing the light intensity can increase the number of photons incident on the CsPbBr_3_ perovskite film, resulting in an increase in the photocurrent. Responsivity refers to the ratio of the photocurrent to the light intensity of the photodetector under irradiation of a specific wavelength and represents the photoelectric conversion efficiency of the photodetector. Responsivity (*R*) can be expressed as follows:

2 $$R=\frac{({I}_{light}-{I}_{dark})}{PS}$$

where *I*_*light*_ and *I*_*dark*_ represent the light and dark current of the photodetector, respectively; *P* represents the light intensity of the light source, and *S* is the active area of the photodetector (1.38 × 10^–2^ mm^2^). The detection sensitivity of a photodetector to light is usually expressed as the specific detectivity (D*), which could be calculated by the following equation (assuming that the noise limiting the detectivity is major contributed by shot noise from the dark current [52]):

3 $${D}^{*} = \frac{R}{{(2e{I}_{dark}/S)}^{1/2}}$$

where *e* is the elementary charge (1.602 × 10^–19^ C). Figure 4f shows the response of the photocurrent (*I*_*light*_–*I*_*dark*_) and the responsivity and specific detectivity of the photodetector to different light intensities of a 520 nm light source at a 6 V bias. Due to the high crystallinity and compactness of the SRP, the photocurrent can be effectively collected by the electrodes, allowing a relatively large photocurrent to be generated under low light intensities. Furthermore, *R* decreases at a higher incident light intensity because more charge recombination is expected in this case [31]. Therefore, the peak responsivity reaches 1.44 × 10^5^ mA W^−1^ at 0.56 μW cm^−2^, and the peak specific detectivity reaches 2.16 × 10^11^ Jones. Figure 4g shows the normalized responsivity curve of the photodetector in the range of 350–700 nm under a 3 V bias. This photodetector has a good responsivity to a light source from 350–530 nm. The responsivity peaks at approximately 520 nm and then decreases with increasing wavelength. This trend is consistent with the absorption spectrum because the CsPbBr_3_ film has a reduced absorption efficiency for photons with a long-wavelength. To evaluate the ability of the photodetector to track the optical signal, the normalized I–t curves is obtained by the photodetector under irradiation with 520 nm pulsed light at optical switching frequencies of 1 Hz, 200 Hz, and 1.5 kHz (Fig. 4g). With the on/off switching of the pulsed light source, the photodetector can quickly and accurately detect the changes in the optical signal and can subsequently generate a pulsed current with the same frequency. As shown in Fig. S12, the − 3 dB cutoff frequency (f_ − 3 dB_) of the device is greater than 1.5 kHz. The response speed refers to the relaxation time needed for the photodetector to convert the incident light signal into a current signal. The time required for the photocurrent to increase from 10 to 90% and the time required for the photocurrent to decrease from 90 to 10% are usually defined as the rise time (*t*_*rise*_) and fall time (*t*_*down*_) of the photodetector, respectively. The *t*_*rise*_ and *t*_*down*_ of the photodetector were 151 and 135 μs in 1.5 kHz, respectively, both of which far exceed the speed of human perception of optical signal. The photodetector is continuously irradiated with pulsed light to investigate its stability and reversibility under air condition (3 V, Fig. 4i). The decay of the device packaged by PDMS is shown in Fig. S13. Due to the natural resistance of the all-inorganic perovskite CsPbBr_3_ film to environmental moisture and oxygen, the device's stable and reversible photoswitching characteristic. Table 1 summarizes and compares the key parameters of the reported perovskite photodetector arrays and the tested conditions and more information refer to Table S3. By comparison, our SRP is ranked highly in terms of its preparation process, responsivity, response time, and detectivity. The excellent comprehensive optoelectronic performance of SRP makes it suitable for mobile detection with rapid changes in optical signals.

**Table 1:** Comparison of key parameters of SPR with reported arrayable photodetectors

Active material arrays	Device structure (mechanism) ^(*)^	Number ^(**)^ of patterning technologies	Effective area [mm^2^] ^(***)^	Response time (τ_r_/τ_d_) [ms] ^(****)^	Responsivity [A W^−1^]	Refs.
(FASnI_3_)_0.6_(MAPbI_3_)_0.4_ film	ITO/SnO_2_/Pe/P3HT/Au (Photodiode)	> 3	4^(^^**^^)^	19/13	—	[34]
MAPbI_3_ nanowire	ITO/Pe/Au (Photodiode)	3	4 × 10^–2^	20.6/13.8	3.6 × 10^–2^	[33]
CsPbBr_3_ quantum dots	Ni-Au/Spiro- OMe TAD/Pe/TiO_2_/ITO (Photodiode)	2	8 × 10 ^−2^	2.3 × 10^3^/—	21.2	[51]
FAPbI_3_ nanowire	W/Ionic liquid/Pe/Ga-In (Electrochemical photodetector)	> 3	0.38	32.0/40.8	0.30	[44]
FA_0.85_Cs_0.15_PbI_3_ film	Au/Pe/Au (Photoconductor)	2	3 × 10^–3^	0.0137/0.0149	4.8	[45]
MAPbI_3−x_Cl_x_ film	Au/Pe/Au (Photoconductor)	2	1 × 10^–2 (**)^	480/260	2.17	[32]
CsFAMAPbIBr film	Ti_3_C_2_T_x_/Pe/Ti_3_C_2_T_x_ (Photoconductor)	2	0.46	24.6/14.7	84.77	[46]
MAPbI_3_ pyramids film	Cu/Pe/Cu (Photoconductor)	2	0.14	0.7/1.1	28.8	[47]
MAPbI_3−x_ Cl_x_ film	Au/Pe/Au (Photoconductor)	3	1.56^(^^**^^)^	25/32	1.21 × 10^–5^	[48]
MAPbI_3_ film	Au/Pe/Au (Photoconductor)	3	6 × 10^–3^	52.7/57.1	2.83	[49]
MAPbX_3_ film	Au/Pe/Au (Photoconductor)	> 3	2.2 × 10^–5^	65/68.2	14.97	[50]
CsPbBr_3_ nanoplates film	Ti_3_C_2_T_x_/Pe/Ti_3_C_2_T_x_ (Photoconductor)	2	2.1^(^^**^^)^	48/18	0.45	[30]
CsPbBr_3_ film	Au/Pe/Au (Photoconductor)	2	1 × 10 ^−2 (**)^	8/6.5	3.15	[25]
CsPbBr_3_ film	ITO/Pe/ITO (Photoconductor)	1	1.38 × 10^–2^	0.151/0.135	144	This work

^(*)^ Pe in this column denotes perovskite

^(**)^ Detailed descriptions and statistics of the technologies are shown in Table S3

^(***)^ This area refers to the active area of the photodetector unit. If the effective irradiated area was not specified in the article, then it was calculated based on the area of the photodetector unit

^(****)^ The frequency of light source reference Table S3

### Applications of Photodetector Array in Non-contact Human–Machine Interaction

**Fig. 5:**
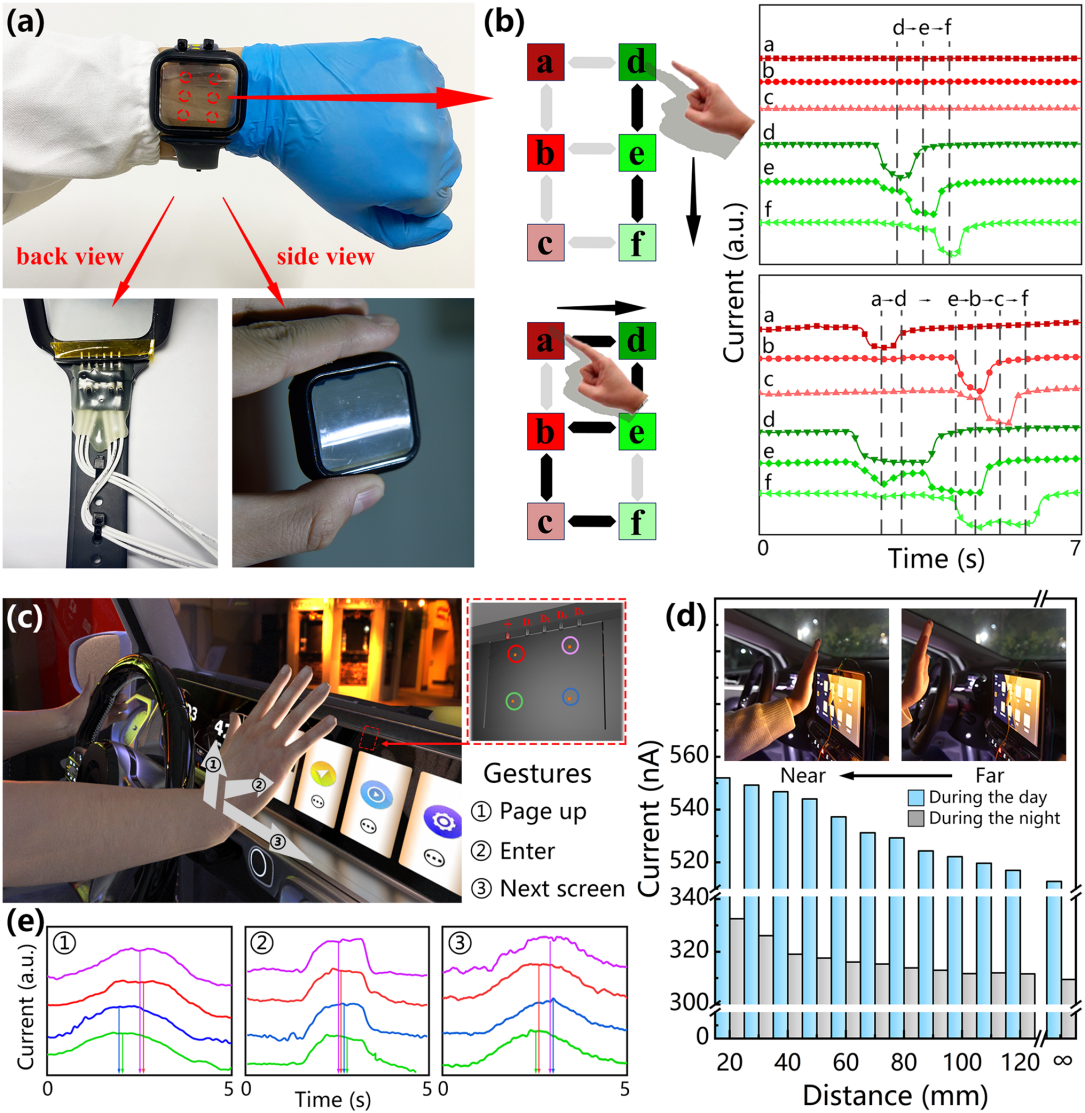
Application of the SPR array for non-contact human–machine interaction in flexible wearable devices and integrated 3D gesture detection. **a** The photograph of the flexible wearable device. **b** Gesture writing of the numbers “1” and “2” and the corresponding current. **c** The 3D gesture detection of the human–machine interface on the integrated car screen. **d** Schematic diagram of the detection mechanism of 3D motion detection and the current with the distance between the human hand and the display under a 5 V bias. **e** The current for different gestures at a 5 V bias

Figure 5A shows a photograph of the flexible wearable device. A polyethylene terephthalate (PET) flexible film was used as the substrate of the device, which provided the device with excellent transparency and appropriate stiffness so that the device can work stably on the curved surface like a smart watch. We integrated external circuitry for data acquisition on the back of the silicone strap, inserted the metal probe into the custom-made watch case, connected the probe with the flexible interface inside the case through a good electrical contact, and finally insulated and fixed the connection with polyimide tape. Figure S14 shows the current of the flexible device at different bending angles. This device can maintain a relatively stable photoelectric properties at a certain bending angle, so the use of the device is not affected in a fixed-angle scenario (such as curved-screens and wearable devices). Figure S15 shows the SEM image of the split-ring structured perovskite film on the ITO–PET substrate, and the crystalline film is still of high quality, which fully demonstrates the universality of the preparation method. A segment code liquid crystal display (LCD) display generally uses the visible states of seven-line segments to represent the numbers 0 to 9. Owing to this display method has simple programming and low power consumption, it has been widely used in LCD digital displays. According to the display mode of the segment code LCD displays, a total of six connection points or corner points are generated between the seven-line segments to express characters of any number. Hereby, we can determine the movement of the finger by detecting the spatiotemporal series in the photocurrent of the photodetector array, and the circuit diagram of this interface is shown in Fig. S16. Figure 5b shows the input gestures for “1” and “2” and the corresponding current data of the photodetector array. When a person writes numbers on the wearable device, the current of the photodetector decreases rapidly due to the shading of the finger. As the finger continues to move, the shadow leaves the photodetector site, and the photon irradiation causes the photodetector to rapidly recover to the initial current level. By using valleys in the I-t curve of the six photodetector units to judge gesture actions, which is beneficial to reduce data errors generated by other photodetector sites during the writing process. The SRP array spacing is 1.5 cm, this suitable spacing makes the writing gesture more comfortable for humans and can improve the accuracy of input signals. By writing the numbers "0, and 3–9," the measurement program of the photodetector can obtain the changes in the current data of different time series (Fig. S17). The realization of this function will help humans to input more instructions and information on the human–machine interface.

The automobile has greatly changed the way that people travel, and the car display has become an important branch of human–machine interaction. The operation interface of the automobile is designed to focus on the safety of the driver. We have innovatively developed a photodetector array-based 3D gesture detection scheme (Fig. 5c). By integrating a transparent device with the car display of the automobile, human can use simple touchless gestures during driving to achieve safe and comfortable interactions with the car. This interactive system can realize the recognition of 2D swiping gestures (for instance, swiping up and right are defined as page-up and next screen, respectively) and even 3D gestures (for instance, moving inward along the normal line of the screen is defined as “enter”). Figure 5d illustrates the application of the interactive system on 3D gestures motion in different driving environments, including day-time and night-time by testing the current data corresponding to different distances between the hand and the screen. Figure S18 shows a photograph of this test platform. When the hand approaches the device from a far distance, the current of the photodetector shows a gradually increasing trend. The current increases when the hand is 120 mm away from the screen and reaches its peak value when the hand is 20 mm from the screen. The current increased by 7.65% under the day-time and by 7.43% under the night-time driving condition, indicating that this device has high spatial perception and sensitivities under different lighting conditions. The detection mechanism of the 3D gesture is shown in the inset of Fig. 5d. A portion of the photons emitted by the display will be directly incident on the device so that the photodetector reaches a stable photocurrent level, and the remaining large number of photons will pass through the transparent substrate of the photodetector and radiate outward. When the human hand approaches the display, the skin surface will diffusely reflect the light. The photons reflected by the skin are irradiated to the photodetector, and further increasing the current. At the night-time, current growth rate is equivalent to that in the day-time driving, due to the reduction in the number of ambient light sources still helps to improve the response of the photodetector to the light reflected by the hand surface. Figure 5e shows the current data of the gestures of moving upward, forward and left, and refer to Fig. S16 for the circuit diagram of the system. The sequence that the current reaches its peak value can be used as the spatiotemporal series for determining the direction of the gesture movement. After the gesture passes through the photodetector array, the movement direction of the gesture can be perfectly matched based on the graphs, which demonstrates the 3D motion sensing capability of the integrated non-contact human–machine interface and provides a new insight into the sensing display-integrated multifunctional interactive system.

**Fig. 6:**
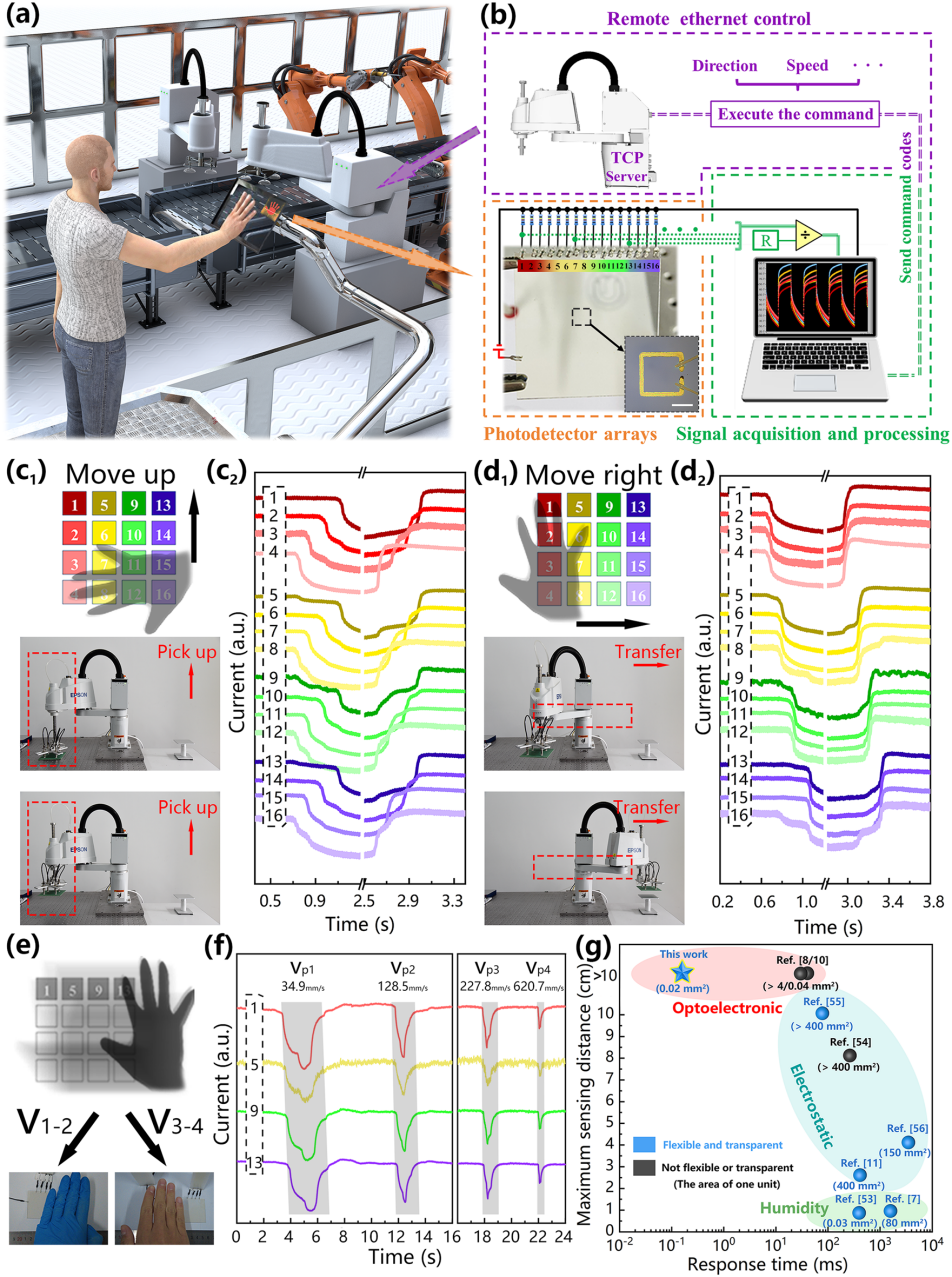
Remote control of robot based on gesture interaction. **a** The schematic diagram of the non-contact human–machine interaction scenario. **b** The schematic of the human–machine interactive system, including the construction of the photodetector array, real-time data acquisition and processing, and remote Ethernet control. **c**_**1-2**_ The robot’s picking up process. **d**_**1-2**_ The robot’s transfer process. **e** The schematic diagram of the real-time detection of gestures at different moving speeds by the device. **f** *I-t* curves of photodetector arrays at a series of moving speeds. **g** Comparison of sensor parameters of our device with reported sensor array for non-contact gesture detection

Our photodetector-based interactive system is expected to enable more interactive functions between humans and machines, instead of only signal input and information transmission. In many application scenarios (such as automated factories that require high-temperature reactions, laboratories that store corrosive reagents), direct contact between the operator and the machine should be avoided as much as possible. Figure 6a schematically depicts a scenario of a factory, in which humans can control the robot in real time through a touchless system. Figure 6b shows a schematic diagram of the circuit setup of the system. This construction is divided into three parts: the external circuit connection of the photodetector array, the acquisition and processing of real-time data, and the remote Ethernet control of the robot. We use a 4 × 4 array of photodetectors helps to improve the accuracy of program judgment and the complexity of operating commands. The sequence and interval of the shading of different photodetector units can be mapped to the movement direction and speed of the hand shading the photodetector. Hence, when the current drops below the threshold, the program will record and logically determine the identifier number of the photodetector unit, and then will output the data as the control command code that can be recognized by the robot controller. The real-time remote control of the robot must be completed by using LabVIEW as the client of the transmission control protocol (TCP) to send the control command code to the TCP server in the robot. We obtain on/off current data for 16 photodetector units under illumination by light-emitting diode (LED) and hand shaded (2 cm from the photodetector), respectively (Fig. S19). The standard deviations of the current and dark current of the photodetector arrays are 9.02 × 10^–8^ and 1.16 × 10^–8^, and the average on/off ratio is approximately 13.06, which can effectively reduce the program judgment errors. Figure 6c_1_ shows the manipulation of the sample grasping orientation by the robot through gestures. First, a human uses the swiping-up gesture from the bottom row of the photodetector array (4, 8, 12, 16) to the top row (1, 5, 9, 13). With the movement of the hand, the photodetector is exposed to the light source again. The real-time photocurrent variations are shown in Fig. 6c_2_. The current in each column drops almost simultaneously, while the current in each row gradually decreases. Figures 6d_1-2_ shows a robot performing sample transfer under remote control. When the hand laterally swipes over the photodetector array, the current in each column gradually decreases, while the current in each row changes simultaneously. The human–machine interaction process described above, including the robot's picking and transferring of targets, is shown in Video S3. The non-contact control process is accurate and fast, taking full advantage of the unique advantages of this system. Figure 6e demonstrates the speed detection function of the human–machine interface. A bare right hand and a gloved left hand move at different speeds in front of a row of photodetector arrays. Gestures with different movement speeds (V_1_-V_4_) are detected using two measurement methods, namely imaging-based measurement (M_i_) and photodetector-based measurement (M_p_). The detection mechanism of the imaging-based measurement method is to record the number of video frames corresponding to the gesture at different mark positions and convert the number of frames into time to calculate the movement speed of the gesture. The movement speeds measured by this method were V_i1_ = 30.2 mm s^−1^, V_i2_ = 109.2 mm s^−1^, V_i3_ = 240.4 mm s^−1^, and V_i4_ = 600.0 mm s^−1^. The speeds measured by the photodetector-based measurement method were V_p1_ = 34.9 mm s^−1^, V_p2_ = 128.5 mm s^−1^, V_p3_ = 227.8 mm s^−1^, and V_p4_ = 620.7 mm s^−1^. The speeds detected by the two methods are approximately the same. The current curves and the corresponding real-time video records at different gesture speeds verify the extremely high sensitivity and accuracy of the photodetector-based human–machine interface in the speed detection of moving objects (Fig. 6f and Video S4). To further compare the existing touchless gesture detection technologies, we summary the key sensor parameters of these technologies in Fig. 6g [7, 8, 10, 11, 53–56]. The photodetector-based interface we developed is superior in transparency, flexibility, detection distance, and response speed.

## Conclusion

In summary, we studied systematically the split-ring topography from wettability, evaporative assembly to optoelectronics. The split-ring lyophilic patterns and electrode arrays were fabricated by a dual-function laser etching technique simultaneously. Compared to the fully lyophilic and square ring patterns, the split-ring pattern can capture 82% of precursor solution while the deposition area was reduced to 38%. This scheme not only assisted the highly efficient directional transportation of liquid, high-throughput fabrication of perovskite arrays and high compactness of perovskite film, but also simplified the preparation process and reduced the cost of the devices. Due to the high compactness and excellent optoelectronic properties of the split-ring structured, the photodetector we developed has the maximum responsivity reached 1.44 × 10^5^ mA W^−1^, the maximum on/off ratio reached 8.2 × 10^3^, and the response time reached 150 μs in 1.5 kHz.

We successfully implemented the human–machine interaction, including writing numbers, gesture detection, in scenarios such as wearable devices, automobile displays and remote control, fully demonstrating the potential of our non-contact scheme based on photodetector arrays in various environments. However, in practical applications, gesture detection is affected by many factors, such as the position of the light source, personal gesture habits, reflections or shadows of other objects, and so on. Therefore, further development of artificial intelligence assisted, or trained signal analysis software is necessary to reduce misjudgment and obtain an accurate spatiotemporal series. Moreover, the lyophilic-lyophobic patternsassisted dewetting approach enables a new fabrication technology with high-throughput of large-scale split-ring array. Given the featured size in the samples, the split-ring meta-structure can be readily developed into terahertz metamaterials [57–59]. Therefore, our results will also hold the promising application toward the structural design and preparation of metamaterials and their active application in the field of terahertz metamaterials.

### Supplementary Information

Below is the link to the electronic supplementary material.

Supplementary file1 (MP4 1231 KB)

Supplementary file2 (MP4 1969 KB)

Supplementary file3 (MP4 476 KB)

Supplementary file4 (MP4 174 KB)

Supplementary file5 (DOCX 6607 KB)
