# Supplementary material for: Split-Ring Structured All-Inorganic Perovskite Photodetector Arrays for Masterly Internet of Things
Source: Nanomicro Lett. 2022 Nov 29;15:3. doi: 10.1007/s40820-022-00961-y (PMC9709000; doi:10.1007/s40820-022-00961-y)
Supplement: Supplementary file 5 — Supplementary file5 (DOCX 6607 KB) [file 40820_2022_961_MOESM5_ESM.docx]

**Supplementary Information**

**Split-Ring Structured All-Inorganic Perovskite Photodetector Arrays for Masterly Internet of Things**

Bori Shi^1^, Pingyang Wang^1^, Jingyun Feng^1^, Chang Xue^1, 2, 3^, Gaojie Yang^4^, Qingwei Liao^5^ Mengying Zhang^6^, Xingcai Zhang^4, 5, 7*^, Weijia Wen^3, 8^, Jinbo Wu^1, 2, 3*^

^1^Materials Genome Institute, Shanghai University, Shanghai 200444, China.

^2^Zhejiang Laboratory, Hangzhou 311100, China.

^3^HKUST Shenzhen-Hong Kong Collaborative innovation Research institute, Futian, Shenzhen China.

^4^School of Engineering, Massachusetts Institute of Technology, Cambridge, MA, 02139, USA.

^5^School of Engineering and Applied Sciences, Harvard University, Cambridge, MA, 02138, USA.

^6^Department of Physics, Shanghai University, Shanghai 200444, China.

^7^Brigham and Women's Hospital, Harvard Medical School, Boston, MA, 02115, USA.

^8^Advanced Material Thrust, The Hong Kong University of Science and Technology (Guangzhou), Guangzhou, China.

* Xingcai Zhang. E-mail: xingcai@mit.edu; * Jinbo Wu. E-mail: [jinbowu@t.shu.edu.cn](mailto:jinbowu@t.shu.edu.cn)


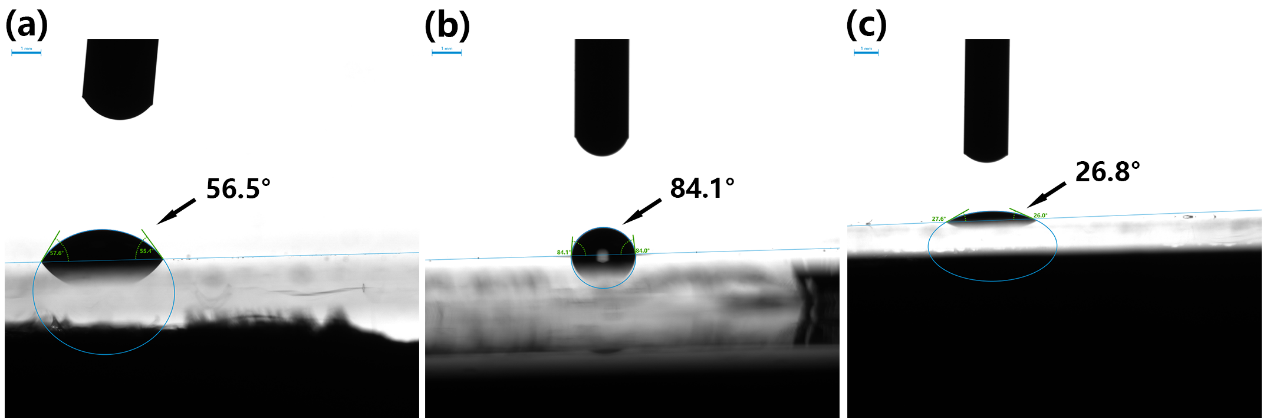


**Fig. S1** Contact angles of different substrates to perovskite precursors. **a** ITO-glass. **b** lyophobic substrate treated by POTS. **c** Laser-etched ITO-glass

**Table S1** The adhesion energy of different surfaces. $\theta$ is the contact angle between diiodomethane and the surfaces, $\sigma_{S}^{D}$ is the dispersive component of the surface energy of the surface, $\sigma_{S}$is the total surface energy of the solid. $I_{SL}$ is the adhesion energy between DMSO and solid surface in unit area

**Calculation of the adhesion energy of DMSO**

Theoretically, the Fowkes Theory describes the surface energy of a solid as having two components (a dispersive component and a polar component). Mathematically, Fowkes Theory is based on three fundamental equations which describe the interactions between a solid surface and liquids. These equations are as follows:

Young’s Equation: $\sigma_{S}=\sigma_{SL}+\sigma_{L}\text{cos}\theta$ (1)

wherein: $\sigma_{L}$ is the total surface tension of the wetting liquid, $\sigma_{S}$ is the total surface energy of the solid, $\sigma_{SL}$ is the interfacial tension between the solid and liquid, and $\theta$ is the contact angle between the liquid and solid,

Dupre’s Definition of Adhesion Energy: $I_{SL}=\sigma_{S}+\sigma_{L}-\sigma_{SL}$ (2)

wherein: $I_{SL}$is the adhesion energy between a liquid and a solid surface in per unit area .

Fowkes’ Theory’s Definition of Adhesive Energy:

$I_{SL}=2[(\sigma_{L}^{D})^{1/2}(\sigma_{S}^{D})^{1/2}+(\sigma_{L}^{P})^{1/2}(\sigma_{S}^{P})^{1/2}]$ (3)

wherein: $\sigma_{L}^{D}$ is the dispersive component of the surface tension of the wetting liquid, $\sigma_{L}^{P}$ is the polar component of the surface tension of the wetting liquid, $\sigma_{S}^{D}$is the dispersive component of the surface energy of the solid, $\sigma_{S}^{P}$ is the polar component of the surface energy of the solid.

By combining these three equations, the primary equation of the Fowkes' surface energy theory is obtained :

$\left( \sigma_{L}^{D} \right)^{1/2}\left( \sigma_{S}^{D} \right)^{1/2}+\left( \sigma_{L}^{P} \right)^{1/2}\left( \sigma_{S}^{P} \right)^{1/2}=\sigma_{L}\left( \cos\theta+1 \right)/2$ (4)

In equation(4), $\sigma_{L}^{D}$_DMSO_= 34.9 mN/m，$\sigma_{L}^{P}$_DMSO_= 8.6 mN/m, the $\sigma_{S}^{D}$and $\sigma_{S}^{P}$ are unknown. Therefore, to obtain $\sigma_{S}^{D}$ and $\sigma_{S}^{P}$, the test of the adhesion energy is divided into two steps.

The first step in determining the solid surface energy is to test the solid for contact angle $\theta$ with the diiodomethane，which has only a dispersive component to its surface tension (for the diiodomethane, the $\sigma_{L}$=$\sigma_{L}^{D}$= 50.8 mN/m). In this case, the equation (4) reduces to

$\sigma_{S}^{D}=\sigma_{L}\left( \cos\theta+1 \right)^{2}/4$ (5)

$\sigma_{S}^{D}$can be calculated by substituting the contact angle data into the equation (5).

The second step is to test the solid for contact angle with the solvent liquid DMSO. Knowing the liquid's surface tension components and the $\sigma_{S}^{D}$, $\sigma_{S}^{P}$as the only unknown in the primary equation (3) can be calculated as

$\sigma_{S}^{P}+\sigma_{S}^{D}=\sigma_{S}$ (6)

Combine these dates into equation (3), the different adhesion energy can be calculated and shown in table S1.

**Table S2** The properties of glycerol (40 wt%) and DMSO. To reduce the viscosity of glycerol, we prepared glycerol-water solutions in 40% mass fractions

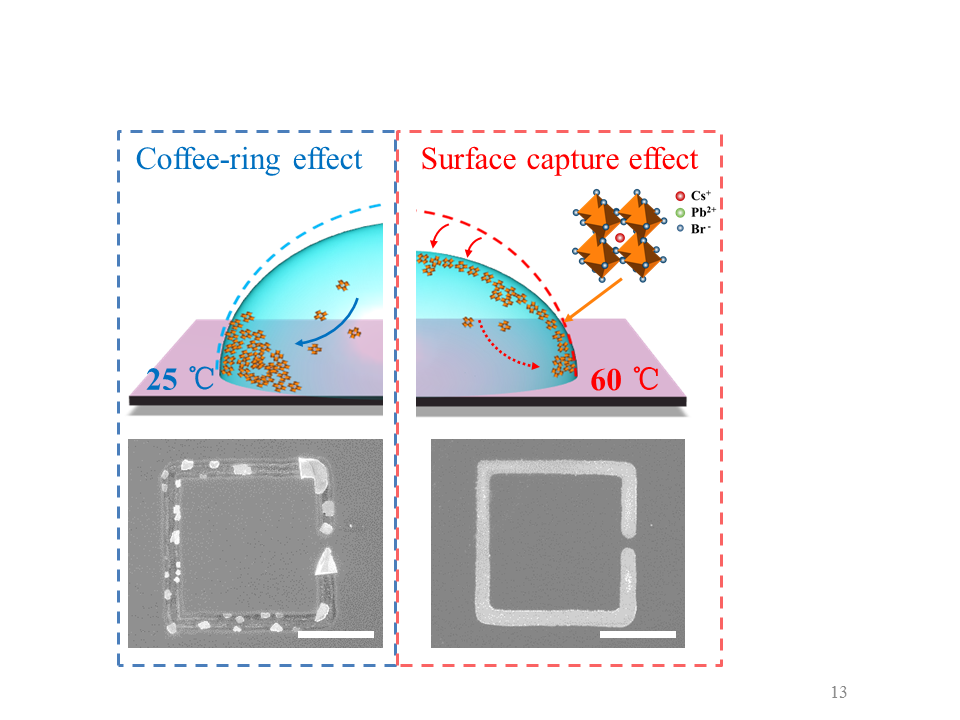


**Fig. S2** Evaporation mechanism and crystallization morphology at room temperature and 60℃ (Scale bar: 100 μm)

At room temperature, due to the slow evaporation process, small crystal nuclei have enough time to grow, and the driving force was dominated by capillary flow that transported nanocrystals to the edge of the pattern. Therefore, a random distribution phenomenon was formed with several large crystals. By comparing the evaporation at 60℃, it can be found that the density of perovskite nanoparticles is significantly increased under the dominance of surface capture effect. Therefore, in our subsequent experiments, the evaporation temperature of 60℃ was adopted to suppress the coffee ring effect and ultimately avoid the formation of large crystals.


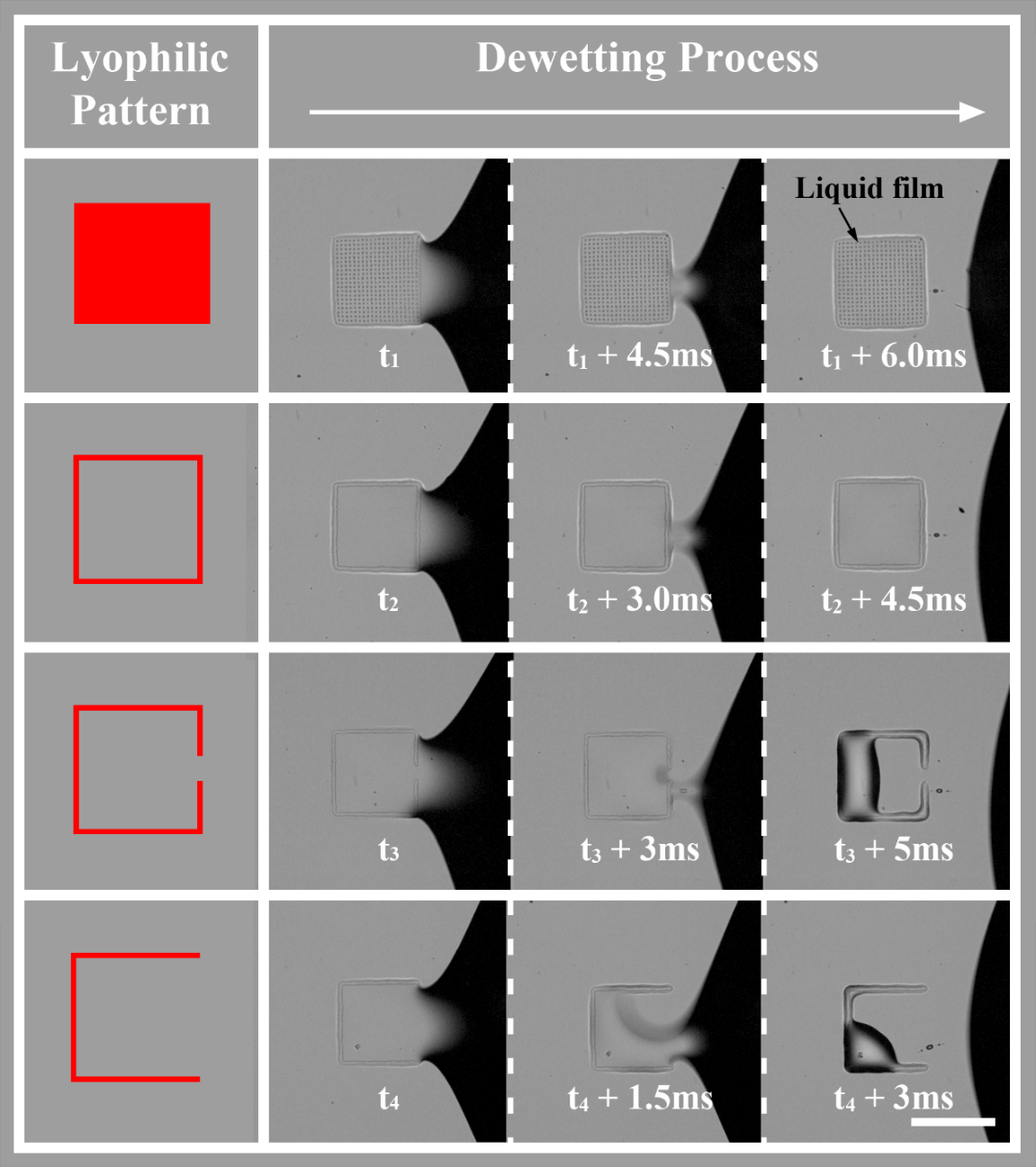


**Fig. S3** Comparison of the dewetting process of the four lyophilic patterns (scale bar: 200 μm)

These four patterns are fully lyophilic square, square ring without opening, split-ring and trilateral pattern. These patterns have the almost same profile, but they have different dewetting performances. The fully lyophilic and square ring pattern capture liquid thin films in the first dewetting because of their closed lyophilic outline. While the split-ring pattern can capture a liquid thin film as well, and the liquid film immediately shrank to a droplet due to imbalance between the pinning force and surface tension. When lyophobic gap width is equal to side length, the split-ring pattern become to be the trilateral pattern. As shown in Figure S3, during dewetting, the three-phase contact line of the liquid bridge shrank along trilateral pattern and left a small droplet in one corner.


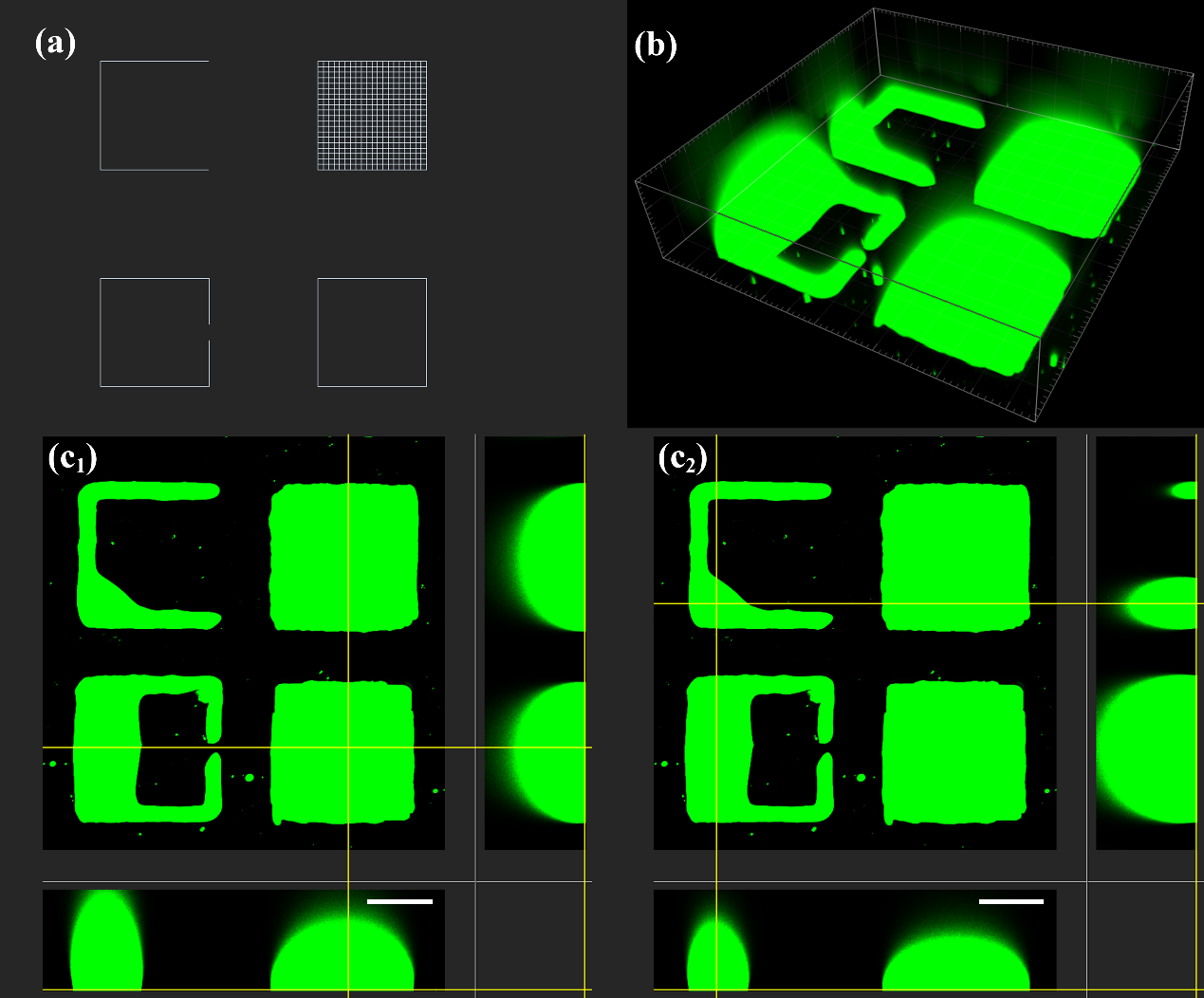


**Fig. S4** The confocal micrographs of glycerol droplets with four patterns. **a** Laser etching drawings of four patterns. **b** The 3D view of four glycerol droplets. **c_1-2_** The top view and cross-sectional of droplets at different locations (scale bar: 100 μm)

We chose 40 wt% glycerol-water solutions with fluorescein sodium salt (2 mM/L) to take confocal micrographs, and then simulated the three-dimensional morphology of perovskite precursors with different patterns at the scale of 200 μm side length. The width of the lyophilic side was measured to be 24 μm. We measured the effective area of each optical section with 1 μm height and then the total volume of one droplet was equal to the product of total effective area and 1μm. The volume of the trilateral / split-ring / square ring / fully lyophilic square of droplets is calculated to be: 0.9 / 2.8 / 3.4 / 3.4 nL, and the effective deposition area is 14400 / 19056 / 19056~50176 / 50176 μm^2^. In particular, the droplets of the square ring pattern have a probability of the liquid film rupture during the deposition process, due to the lyophobic property inside the pattern. Therefore, it is difficult to control the deposition area of the square ring pattern effectively. To evaluate the deposition ability of the precursor solution on different patterns, we defined deposition efficiency = droplet volume/deposition area. For the split-ring pattern, the deposition efficiency is 211% that of the fully lyophilic pattern, validating the high compactness of the split-ring structured perovskite film.


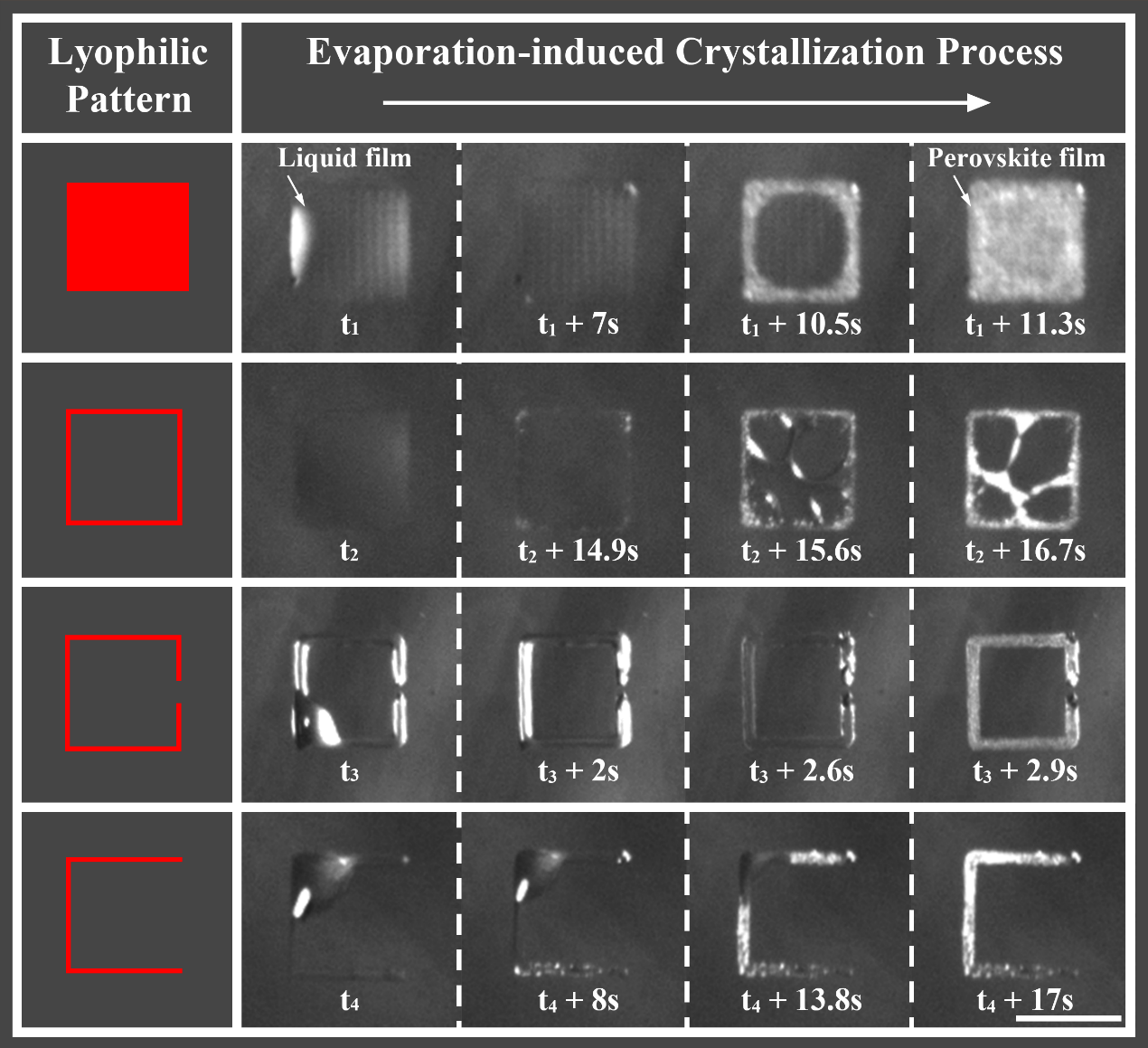


**Fig. S5** Comparison of the evaporation processes of four patterns (scale bar: 200 μm)

In the process of transferring the coated substrate to the 60℃ hot plate and imaging focusing, there will be inevitable time delay, physical vibration and air disturbance. Therefore, the initial state of droplet in this Figure cannot represent the droplet state after coating. The fully lyophilic square pattern, no second dewetting occurs in the evaporation process, has a larger deposition area, and the crystallized film shows poor compactness. As the interior of the square ring pattern is lyophobic, evaporation will increase the probability of liquid film rupture, resulting in random surface deposition. Due to the second dewetting process of the split-ring pattern, precursor solution can be evenly distributed in a relatively small lyophilic region, forming a higher compactness of perovskite film.


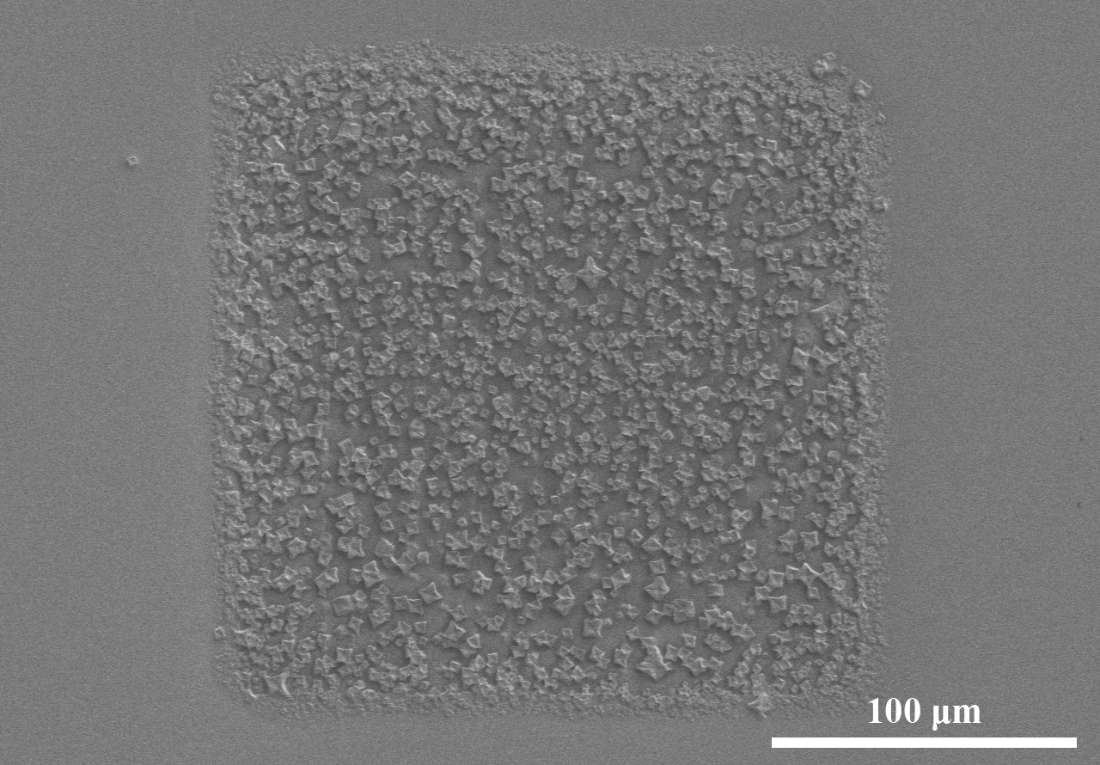


**Fig. S6** The SEM image of perovskite film of fully lyophilic square pattern (scale bar: 100 μm)


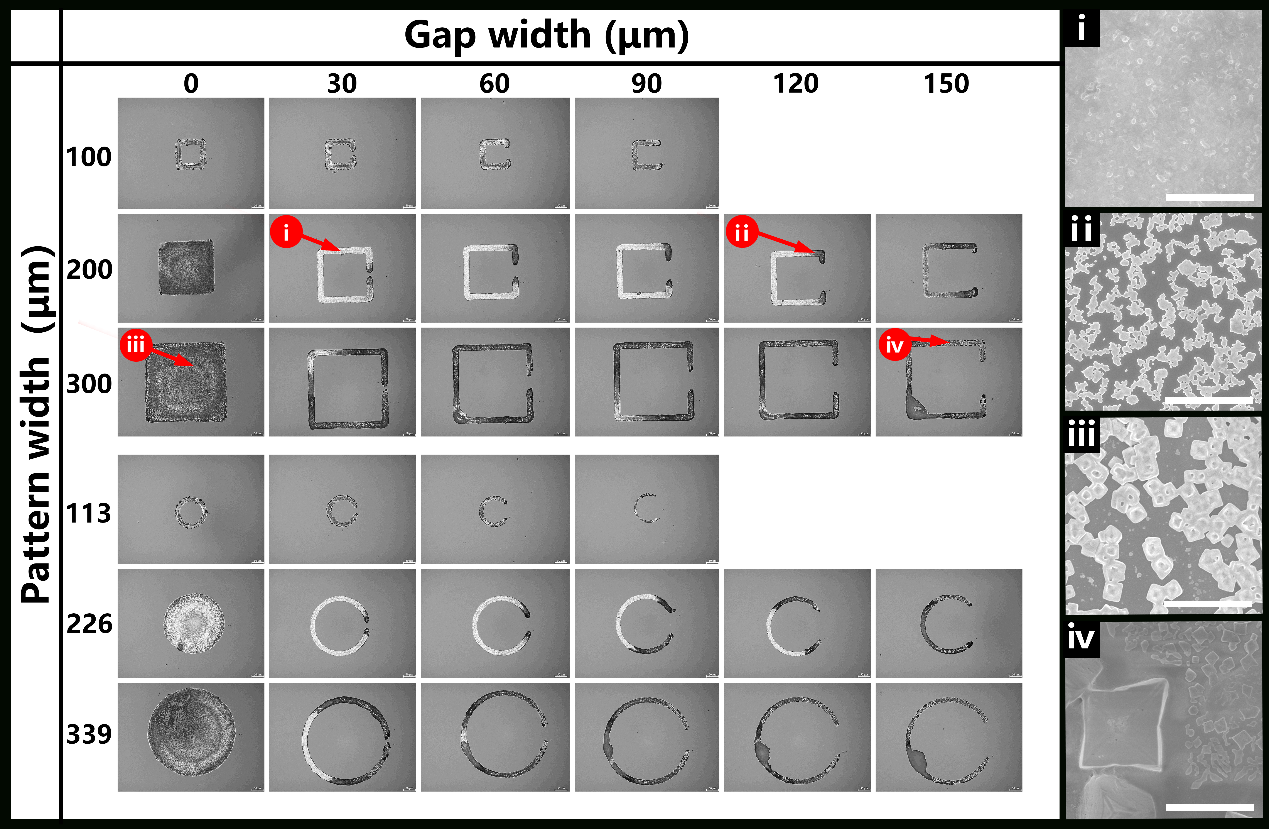


**Fig. S7** Comparison of the different gap widths and pattern width on the perovskite film compactness (scale bar in right: 5 μm)

We compared the effect of pattern width (the width of the square and circular patterns refers to the side length and diameter, respectively) and split gap width (refers to the gap of the laser-etched drawings, the actual gap is smaller than this value, due to the influence of the heat-affected zone) on the film compactness in detail. We found that perovskite films with a side length and gap of 200 and 30 μm had best uniformity and compactness in square patterns. The circular ring pattern with a diameter and gap of 226 and 30 μm had best uniformity and compactness.


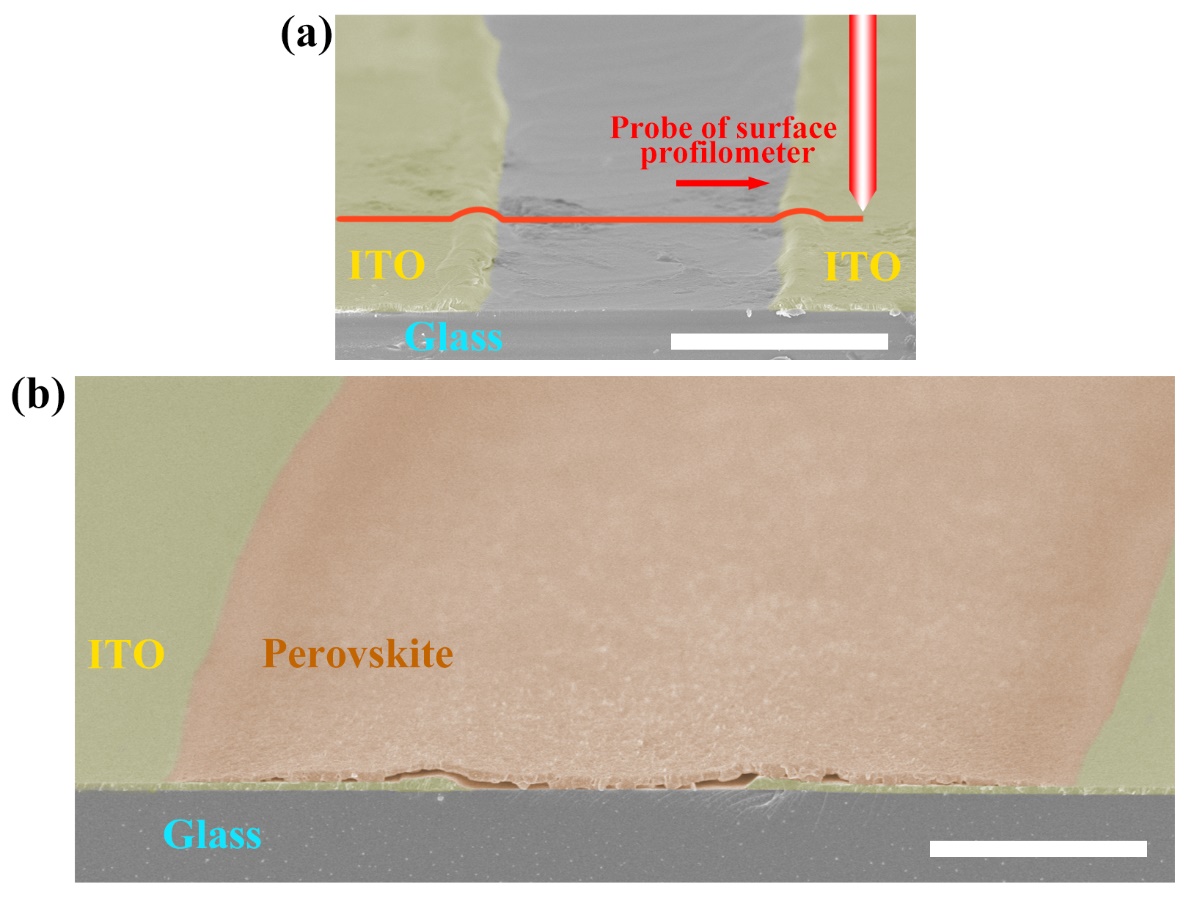


**Fig. S8 a** The schematic diagram of measuring the ITO surface profile. **b** The cross-section SEM of the perovskite photodetector (scale bars: 5 μm)


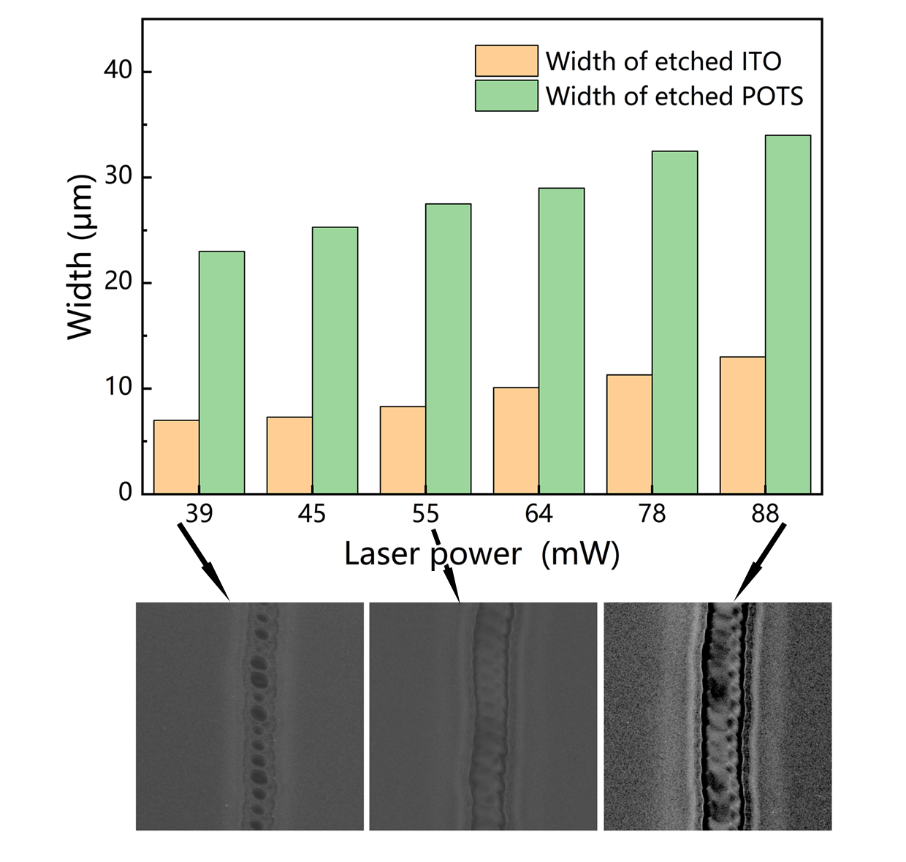


**Fig. S9** Effects of different laser power densities on etch widths of ITO and POTS


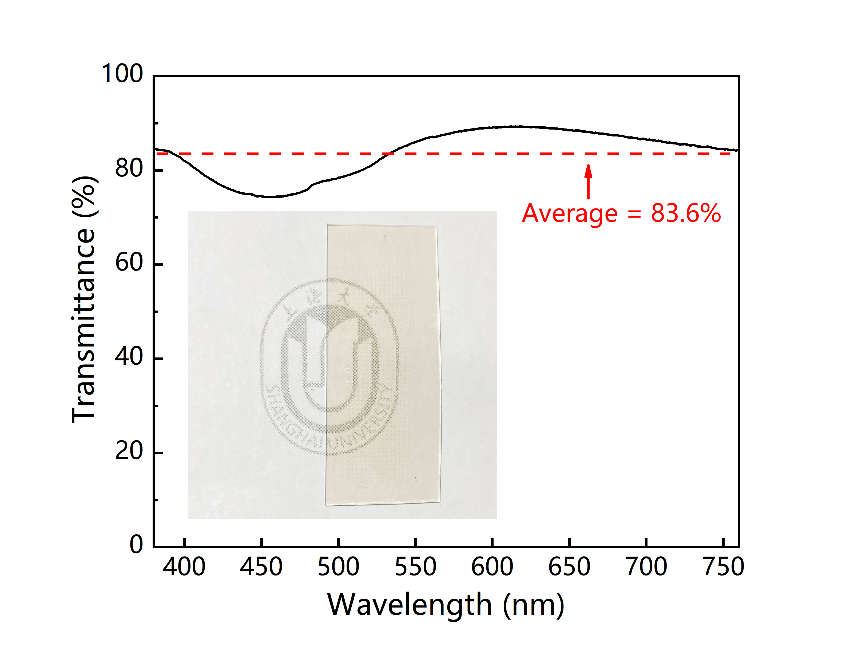


**Fig. S10** The transmittance of the perovskite film array with ITO-glass substrate

**Table S3** Detailed statistics of the arrayable technologies in literature

| Patterning technologies ^(**)^ | Non-patterning technologies | Specific detectivity  [Jones] | Frequency [Hz] ^(****)^ | Bias [V]/  Light source [nm]/  Intensity [μW cm^-2^] | Ref |
| --- | --- | --- | --- | --- | --- |
| Mask radio frequency sputtering, Mask thermal evaporation | Spin-coating | ≈1×10**^11^** | 0.05 | -0.1/850/1.37 ×10^6^ | [34] |
| Anodization process, Mask thermal evaporation | Electrochemical deposition, Vapor-phase growth | ≈1×10**^10^** | 10 | 3/500/— | [33] |
| Mask thermal evaporation, Sliced by scriber | Atomic layer deposition, Spin-coating | 9.35×10^13^ | — | –1.5/405/1.34×10^4^ | [51] |
| Anodization process, Mask thermal evaporation, Mask electrochemical deposition, 3D printing | Vapor-phase growth | 1.1×10**^9^** | 3 | -3/—/0.3 | [44] |
| Photolithography | Chemical vapor deposition, Electron beam evaporation, Spin-coating | 4.2×10**^12^** | 20k | —/650/23.1 | [45] |
| Mask magnetron sputtering, Photolithography | Spin-coating | 9.40×10**^11^** | — | 5/650/33 | [32] |
| Laser etching | Spin-coated | 3.22×10**^12^** | 1 | 3/532/0.24 | [46] |
| Mask thermal evaporation, Nanosphere lithography | Spin-coating | 3.5×10**^11^** | 1 | 5/650/3 | [47] |
| Photolithography, Mask thermal evaporation | Radio frequency sputtering, Spin-casting | 2.44×10**^8^** | 2 | —/—/1.40×10^3^ | [48] |
| Soft photolithography, Mask deposition | Spin-coating | 5.4×10**^12^** | 6k | 3/660/4.57 | [49] |
| Electrohydrodynamic printing, Mask vacuum evaporation, Photolithography | — | 1.41×10**^12^** | 1 | 3/530/100 | [50] |
| Spray-coating, UV cold working | Spray-coating | 6.4×10**^8^** | 0.5 | 10/450/7.07×10^3^ | [30] |
| Photolithography, Mask magnetron sputtering | Vacuum-assisted drop-casting | 3.94×10**^12^** | 1 | 5/—/4 | [25] |
| **Laser etching** | **Sliding method, Magnetron sputtering** | **2.16×10^11^** | **1.5k** | **6/520/0.56** | **This**  **work** |

**^(**)^** The number of technologies refers to the number of patterning (array) technologies, such as laser etching, photolithography, inkjet printing, masked thermal evaporation, and magnetron sputtering. If a technology was used to prepare different materials in different processes, then the technology was counted multiple times. In addition, uniform deposition technologies, such as spin coating, coating, and maskless deposition, were not statistically analyzed.

**^(****)^** Frequency of pulsed light used to test response speed.





**Fig. S11** Logarithmic I–V curves of the photodetector under the irradiation of a 520 nm light source and in the dark


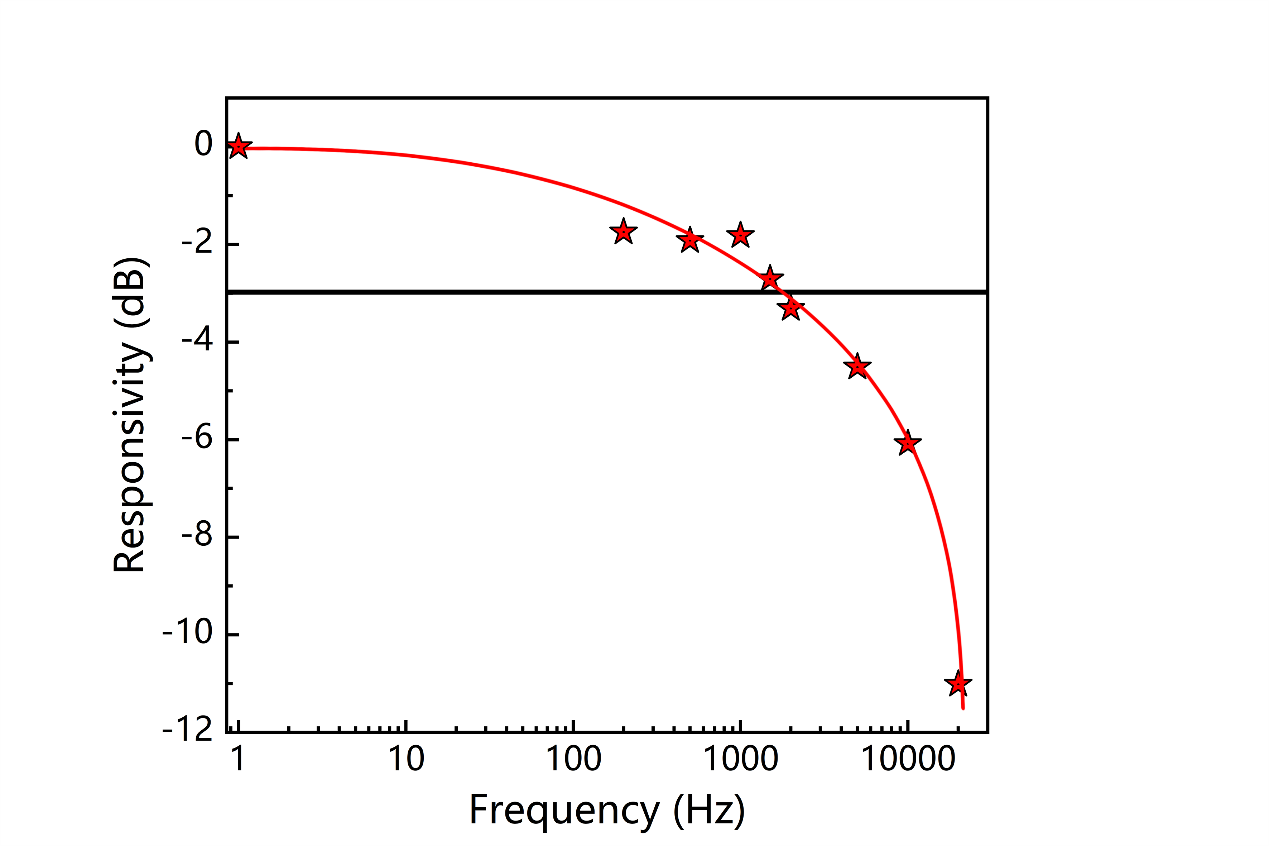


**Fig. S12** Responsivity versus the input signal frequency.





**Fig. S13** I–t curves of the device within 15 days (bias voltage of 3 V and device encapsulated by PDMS have been placed and tested in air environment).


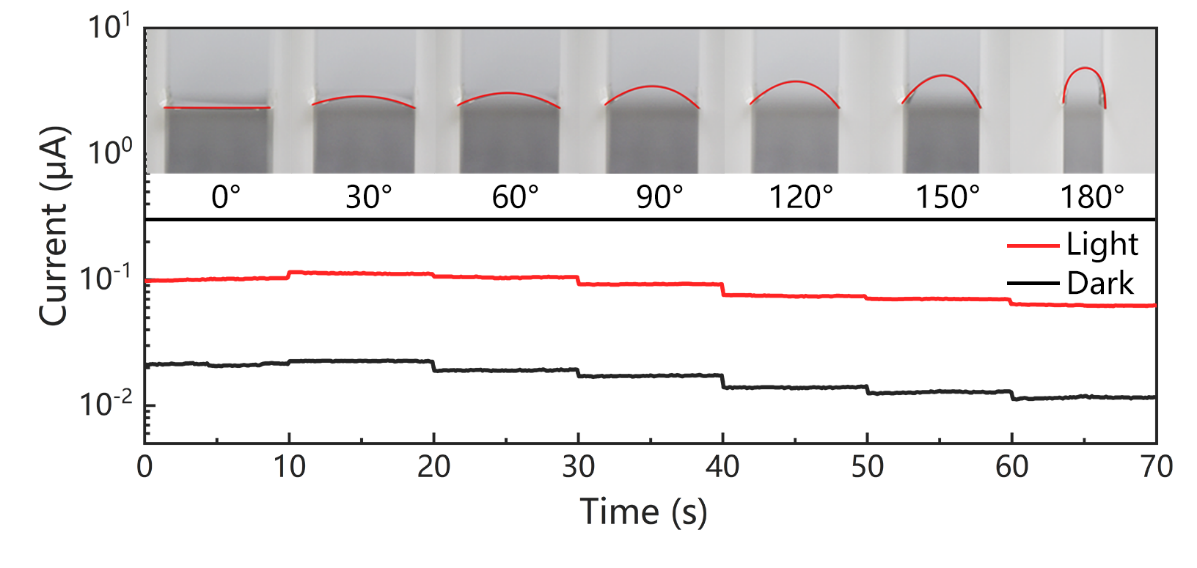


**Fig. S14** I−t curves of photodetector bending at different angles (0°−180°)


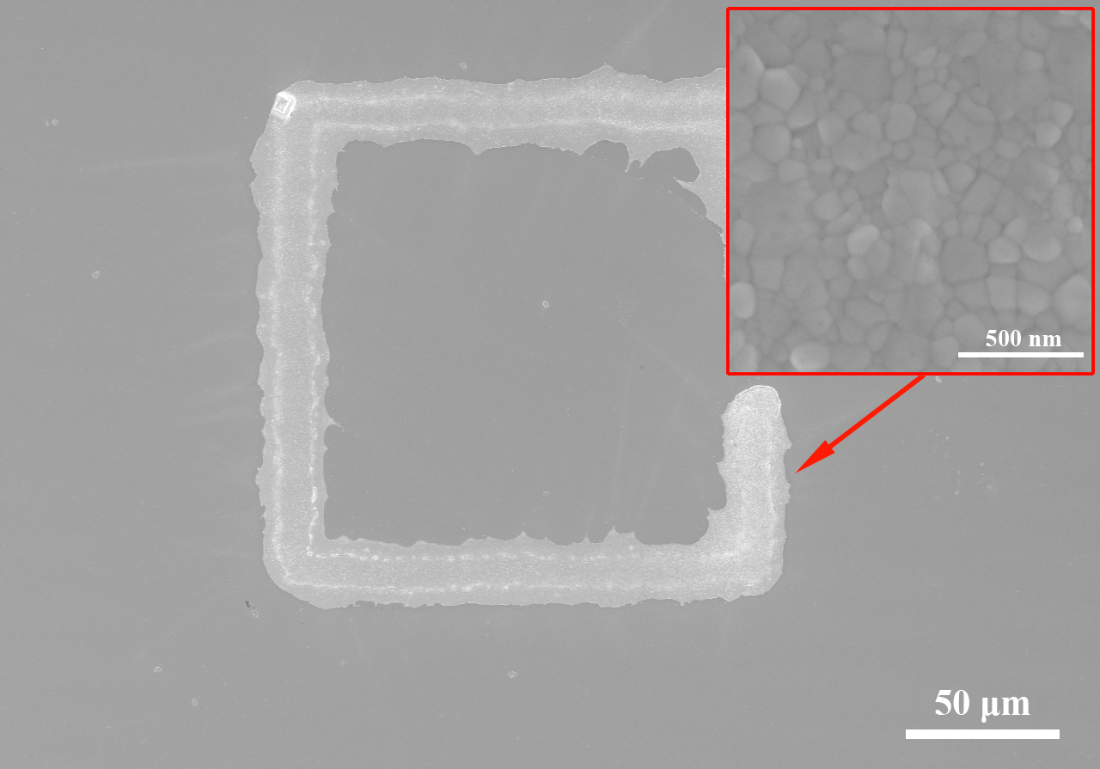


**Fig S15** The SEM image of the perovskite film on ITO-PET substrate


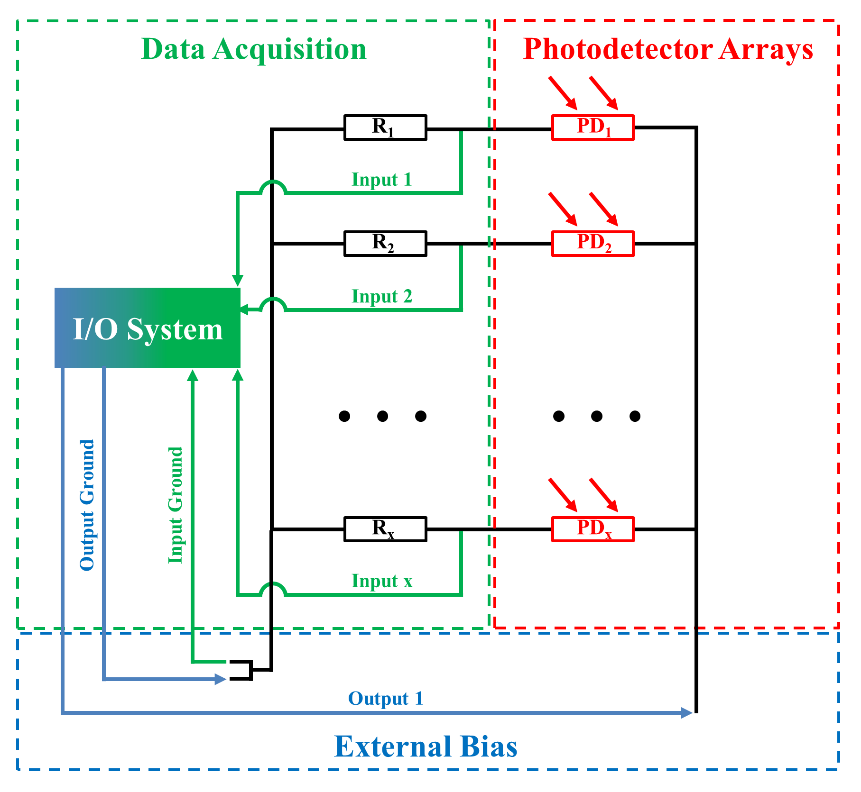


**Fig S16** The circuit diagram of the photodetector arrays


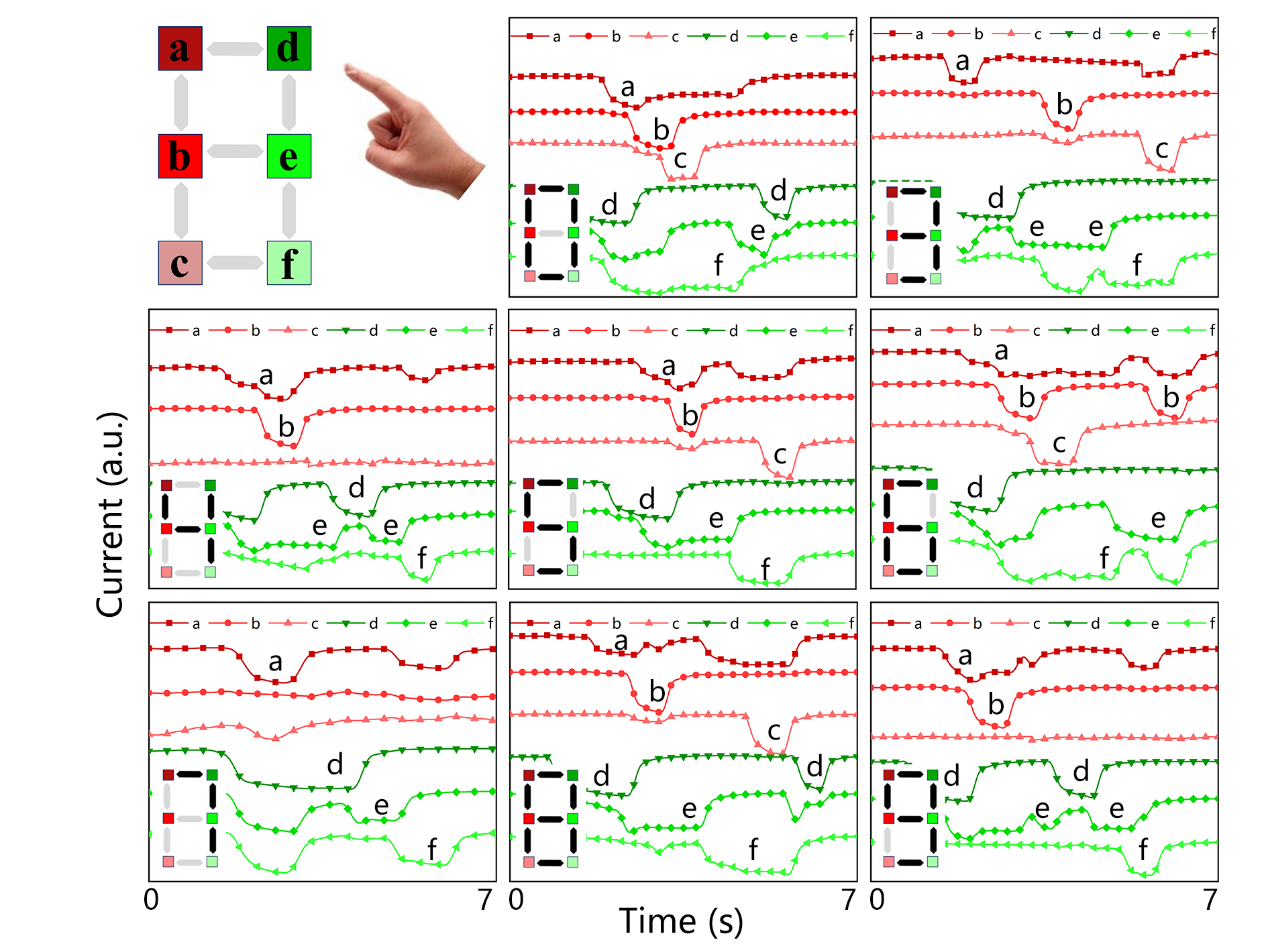


**Fig. S17** I-t curves for writing numbers "0 and 3-9"


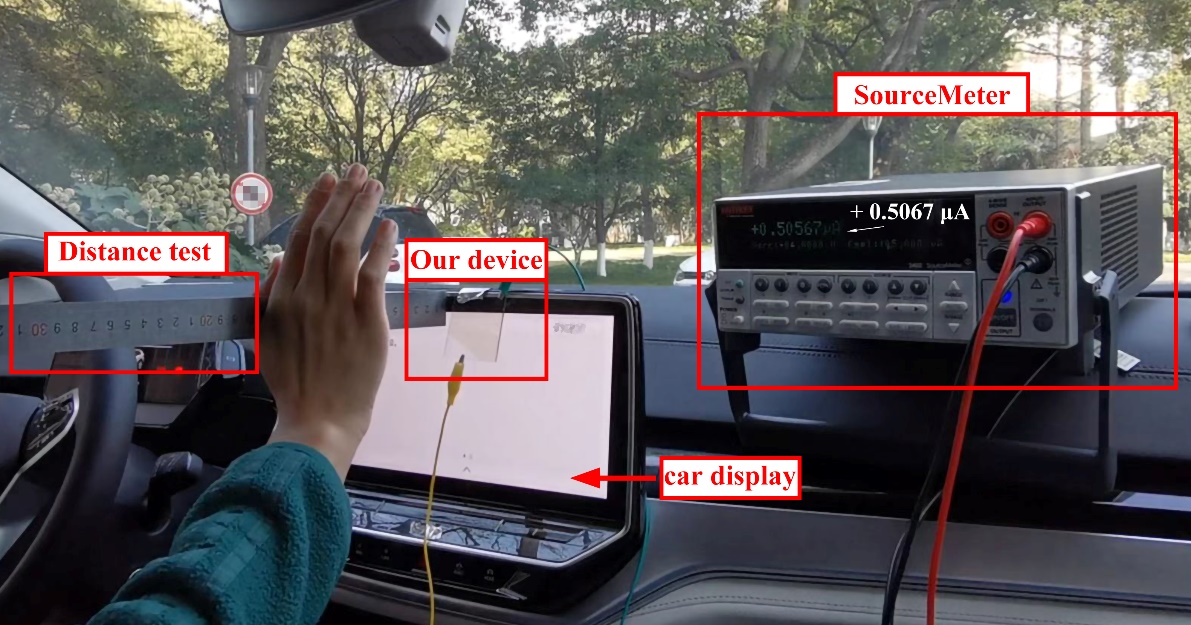


**Fig. S18** The photograph of a 3D exploration platform





**Fig. S19** The current of each photodetector unit under illumination and shading

**References**

[25] W. Wu, X. Han, J. Li, et al., Ultrathin and Conformable Lead Halide Perovskite Photodetector Arrays for Potential Application in Retina-Like Vision Sensing. Adv. Mater. **33**(9), e2006006 (2021). <https://doi.org/10.1002/adma.202006006>

[30] W. Deng, H. Huang, H. Jin, et al., All‐Sprayed‐Processable, Large‐Area, and Flexible Perovskite/MXene‐Based Photodetector Arrays for Photocommunication. Adv. Opt. Mater. **7**(6), 1801521 (2019). <https://doi.org/10.1002/adom.201801521>

[31] W. Deng, J. Jie, X. Xu, et al., A Microchannel-Confined Crystallization Strategy Enables Blade Coating of Perovskite Single Crystal Arrays for Device Integration. Adv. Mater. **32**(16), e1908340 (2020). <https://doi.org/10.1002/adma.201908340>

[32] W. Wu, X. Wang, X. Han, et al., Flexible Photodetector Arrays Based on Patterned CH_3_NH_3_PbI_3- x_Cl_x_ Perovskite Film for Real-Time Photosensing and Imaging. Adv. Mater. **31**(3), e1805913 (2019). <https://doi.org/10.1002/adma.201805913>

[33] L. Gu, M. M. Tavakoli, D. Zhang, et al., 3D Arrays of 1024-Pixel Image Sensors based on Lead Halide Perovskite Nanowires. Adv. Mater. **28**(44), 9713-9721 (2016). <https://doi.org/10.1002/adma.201601603>

[34] Y. Wang, C. Chen, T. Zou, et al., Spin‐On‐Patterning of Sn–Pb Perovskite Photodiodes on IGZO Transistor Arrays for Fast Active‐Matrix Near‐Infrared Imaging. Adv. Mater. Technol. **5**(1), 1900752 (2019). <https://doi.org/10.1002/admt.201900752>

[44] L. Gu, S. Poddar, Y. Lin, et al., A biomimetic eye with a hemispherical perovskite nanowire array retina. Nature **581**(7808), 278-282 (2020). <https://doi.org/10.1038/s41586-020-2285-x>

[46] A. Ren, J. Zou, H. Lai, et al., Direct laser-patterned MXene–perovskite image sensor arrays for visible-near infrared photodetection. Mater. Horiz. **7**(7), 1901-1911 (2020). <https://doi.org/10.1039/D0MH00537A>

[47] X. Xu, W. Liu, Z. Ji, et al., Large-Area Periodic Organic–Inorganic Hybrid Perovskite Nanopyramid Arrays for High-Performance Photodetector and Image Sensor Applications. ACS Mater. Lett. **3**(8), 1189-1196 (2021). <https://doi.org/10.1021/acsmaterialslett.1c00298>

[48] J. Jang, Y. G. Park, E. Cha, et al., 3D Heterogeneous Device Arrays for Multiplexed Sensing Platforms Using Transfer of Perovskites. Adv. Mater. **33**(30), e2101093 (2021). <https://doi.org/10.1002/adma.202101093>

[49] B. Wang, C. Zhang, B. Zeng, et al., Fabrication of Addressable Perovskite Film Arrays for High-Performance Photodetection and Real-Time Image Sensing Application. J. Phys. Chem. Lett. **12**(11), 2930-2936 (2021). <https://doi.org/10.1021/acs.jpclett.1c00521>

[50] Q. Wang, G. Zhang, H. Zhang, et al., High‐Resolution, Flexible, and Full‐Color Perovskite Image Photodetector via Electrohydrodynamic Printing of Ionic‐Liquid‐Based Ink. Adv. Funct. Mater. **31**(28), 2100857 (2021). <https://doi.org/10.1002/adfm.202100857>

[51] K. Shen, H. Xu, X. Li, et al., Flexible and Self-Powered Photodetector Arrays Based on All-Inorganic CsPbBr_3_ Quantum Dots. Adv. Mater. **32**(22), e2000004 (2020). <https://doi.org/10.1002/adma.202000004>
